# Antagonistic Activity of Potentially Probiotic Lactic Acid Bacteria against Honeybee (*Apis mellifera* L.) Pathogens

**DOI:** 10.3390/pathogens11111367

**Published:** 2022-11-16

**Authors:** Aleksandra Leska, Adriana Nowak, Justyna Szulc, Ilona Motyl, Karolina Henryka Czarnecka-Chrebelska

**Affiliations:** 1 Department of Environmental Biotechnology, Lodz University of Technology, Wolczanska 171/173, 90-530 Lodz, Poland; 2 Department of Biomedicine and Genetics, Medical University of Lodz, 5 Mazowiecka Str. (A-6 Building), 92-215 Lodz, Poland

**Keywords:** probiotics, lactic acid bacteria, *Apis mellifera* L., honeybee, honeybee pathogens, antagonistic activity, *Paenibacillus* spp., *Melissococcus plutonius*

## Abstract

Lactic acid bacteria (LAB) are an essential part of the microbiota of the digestive tract of honeybees (*Apis mellifera* L.). Antagonistic activity of 103 LAB strains (isolates from different environments) against 21 honeybee pathogens/opportunistic pathogens (with agar slab method) was screened. The growth of *Paenibacillus* genus was inhibited to the most extent. The highest antagonistic activity was demonstrated by *Lacticaseibacillus casei* 12AN, while the lowest by *Apilactobacillus kunkeei* DSM 12361, a species naturally inhabiting the honeybee gut. LAB isolated from the honeybee environment demonstrated stronger antagonism against pathogens than collection strains. The antagonistic activity of cell-free supernatants (CFSs) from 24 LAB strains against 7 honeybee pathogens was additionally assessed at physiological pH with the microtitration method. The same was determined for selected CFSs at neutralized pH. CFSs with physiological pH showed significantly stronger antibacterial activity than CFSs with neutralized pH. The results confirmed that the mechanism of antimicrobial activity of LAB is acidification of the environment. The obtained results may, in the future, contribute to a better understanding of the antagonistic properties of LAB and the construction of a probiotic preparation to increase the viability of honeybee colonies.

## 1. Introduction

*Apis mellifera* L., the Western honeybee, is one of the most economically valuable pollinators on a global scale. It is estimated that up to 35% of human food consumption depends on the pollination of honeybees [1]. At present, honeybees are exposed to many factors that adversely affect the health of entire colonies. A sudden drop in the viability of honeybee colonies was observed in the winter of 2006/2007, which was later identified as a phenomenon known today as “Colony Collapse Disorder” (CCD) [2]. Due to the enormous importance of these insects for the environment and humans, the decline in the number of honeybees aroused interest and prompted a thorough study of potential triggers of CCD [3]. Factors contributing significantly to this disturbing phenomenon include chemical molecules (e.g., pesticides), parasitic mites (e.g., *Varroa destructor*), viruses, and microorganisms (e.g., *Nosema ceranae*) [4]. The influence of these factors may lead to a significant weakening of the immune system of honeybees and the extinction of the entire colony. Many microorganisms threaten the viability of honeybees, such as bacteria and fungi [4]. Most research and attention are devoted to bacteria, which are the causing agents or secondary contributors of diseases that often lead to the destruction of entire colonies. Known honeybee bacterial pathogens include *Paenibacillus larvae*, *Melissococcus plutonius*, *Paenibacillus apiarius*, *Paenibacillus alvei*, and opportunistic pathogens, e.g., *Serratia marcescens*, *Klebsiella pneumoniae*, and *Klebsiella aerogenes* [5,6,7,8,9,10]. The species of fungi of the genus *Aspergillus* and the yeast-like fungus *Aureobasidium pullulans* also are considered as microorganisms affecting the decline in the number of honeybees [11,12]. Through beekeeping activity, honeybee pathogens can quickly spread throughout the colony, often leading to its complete extinction or the sacrifice of an entire apiary to protect other hives. According to Lindström et al. transmission of disease caused by *P. larvae* can occur even at distances greater than 1 km from clinically diseased colonies [13]. Treatment of diseases caused by honeybee pathogens is extremely limited. Antibiotics affect only vegetative cells and, in addition, residues of chemical substances are detectable later in honey and propolis [14]. Honeybee nest cavities often create ideal conditions for the development of parasites and pathogens. Frequent contact between honeybees via trophallaxis, constant humidity, and temperature favor the existence of many possibilities for the spread of microorganisms inside the colony. Honeybee pathogens first act on a single individual and then on subsequent individuals to successfully reproduce and then disperse to a new host [15]. 

Honeybee microbiota is a complex ecosystem of microorganisms responsible for metabolic functions such as energy and vitamin management, satiety regulation, lipid homeostasis, and the adjustment of glucose levels [16]. It positively influences the immune system by regulating the induction of protective responses against pathogens and stimulates immune responses. The microbiota composition is unique to individuals within the same species [16]. Intestinal bacteria belonging to the honeybee microbiota are transmitted between individuals via trophallactic and oral-fecal transmission. Moreover, the spread of lactic acid bacteria (LAB) is favored by the activity of adult individuals and the consumption of bee bread and pollen [17]. The microbiome controls the development and migration of pathogens, thus avoiding the induction of harmful systemic immune responses, which can alter the composition of the intestinal microbiome of honeybees [18]. The structure of the bacterial community of microbiota can also be an indicator of the health status of honeybees [19].

LAB naturally inhabit the digestive tract of honeybees and have been shown to influence the immune responses of these insects, assimilate nutrients, fight pathogens, and maintain microbiota homeostasis in the gut [20]. Due to the production of various compounds, LAB are known as beneficial bacteria, and some genera (e.g., *Lactobacillus*, *Streptococcus*, and *Pediococcus*) are widely used as probiotics. According to the Food and Agriculture Organization of the United Nations World Health Organization, supported by experts from The International Scientific Association for Probiotics and Prebiotics, the definition of probiotics is “live microorganisms which when administered in adequate amounts confer a health benefit on the host” [21]. When consumed in appropriate amounts, probiotics strengthen the host’s immune system, inhibit the growth of pathogens (through various mechanisms such as adherence to epithelial cells or antagonistic activity) and secrete compounds with antimicrobial properties (e.g., lactic acid) [22]. Probiotic microorganisms should demonstrate the ability to survive in the host’s body and withstand prevailing environmental conditions [23]. Compared to antibiotics, they do not threaten honeybees and leave no chemical compounds detectable in honeybee products [24]. In turn, postbiotics are bioactive compounds secreted by probiotics in the fermentation process [25]. According to the definition given on the experts’ panel in 2021, a postbiotic is “a preparation of inanimate microorganisms and/or their components that confers a health benefit on the host” [26,27]. Postbiotic compounds include, but are not limited to, short-chain fatty acids (SCFAs), functional proteins, metabolites, enzymes, bacteriocins, extracellular polysaccharides (EPS), and microbial cell fractions (e.g., cell walls, cell membranes). Postbiotics can affect the microbiota composition and the host’s immune system, thereby protecting it from infections [28]. The antimicrobial activity of LAB is conditioned by the production of metabolites such as hydrogen peroxide and organic acids (e.g., lactate, propionic, butyrate, or acetate), which decreases the environmental pH, thereby inhibiting the growth of pathogens that cannot tolerate acidic conditions. Lactic acid demonstrates antimicrobial activity against bacteria, and according to Ouwehand & Versterlund, acetic acid inhibits the growth of bacteria and fungi, probably due to its high pKa value of 4.87 [29]. The presence of LAB in honeybee gut microbiota significantly affects the health of these insects, starting from the digestion and fermentation of complex aromatic compounds present in pollen by *Lactobacillus* spp. [30]. *Lactobacillus* strains are also positive for the Ent P2 (enterocin P-like bacteriocin) gene and show a broad spectrum of antagonism against harmful bacteria [31]. Other antimicrobial LAB activities include reducing the mummification of larvae infected with nosemosis by more than 80%, increasing survival after infection with *N. ceranae* by 20%, and reducing *Nosema* spp. spores in hives [30]. According to Zendo et al. LAB produce the bacteriocin kunkecin A, which displays antibacterial activity against *M. plutonius* [32]. LAB also contribute to the treatment of American foulbrood by reducing larvae mortality and in vivo inhibiting the growth of *P. larvae* [30]. Many LAB produce small antimicrobial peptides (AMPs), which contribute to the destruction of pathogen cells by inhibiting cell wall synthesis [33]. Moreover, LAB are also responsible for forming biofilms and detoxification of harmful chemicals (e.g., pesticides) [34].

Combating pathogens with antibiotics may weaken the immune system of honeybees [35]. Due to the frequent supplementation of antibiotics, there is concern about the emergence and spread of antibiotic-resistant microorganisms [35]. Thus, there is a growing need to find natural ways to protect honeybees by supporting their natural microbiota and thereby increasing their resistance to pathogen-induced diseases. In addition, there is currently no in-depth research on the effects of certain microorganisms on honeybee health. Many microorganisms can become opportunistic pathogens when the honeybees’ immune system is weakened by other factors (e.g., pesticides). 

This study focuses on determining the antagonistic activity of LAB also naturally occurring in the environment of honeybees against known honeybee pathogens and opportunistic pathogens. Some of the LAB used in the presented experiments were previously isolated from the honeybee environment and characterized [36]. The effect of LAB metabolites on the inhibition of the growth of honeybee pathogens (at physiological and neutralized pH) was tested to check their postbiotic properties. According to the authors’ knowledge, the topic of LAB antagonistic activity against honeybee pathogens was not thoroughly discussed. So far, no studies have been conducted to determine the effect of the postbiotics (i.e., cell-free supernatants) on the inhibition of the growth of pathogenic microorganisms. 

The LAB has the status GRAS (Generally Recognized as Safe) and QPS (Qualified Presumption of Safety status), which is why these bacteria could be used as a biological control agent for maintaining and supporting the welfare of honeybees. The obtained results in this research may, in the future, contribute to the construction of an ecological preparation that protects the health of honeybees at risk to pathogens and, as a result, improves the sanitary conditions in apiaries.

## 2. Materials and Methods

### 2.1. Chemicals, Vessels, and Other Materials

Tryptic Soy Agar and Broth (TSA and TSB), deMan, Rogosa and Sharpe (MRS) broth and agar, Plate Count Agar (PCA), Malt Extract Agar (MEA), Yeast Extract Glucose Chloramphenicol agar and broth (YGC), Wort Broth and Brain Heart Infusion (BHI) broth, fructose and cysteine-hydrochloride, sodium chloride (NaCl), and sodium hydroxide (NaOH) were purchased from Merck Life Science, Warsaw, Poland. Anaerobe Basal Broth (ABB) and AnaeroGen^TM^ Atmosphere Generation Systems sachets were purchased from Thermo Fisher Scientific, Waltham, MA, USA. Cryobanks™ were from Copan Diagnostics Inc., Jefferson Avenue Murrieta, Murrieta, CA, USA. In addition, 96-well U-bottom transparent plates were from Greiner Bio-One GmbH Kremsmünster, Austria. Syringe filters (0.22 µm pore size) were purchased from Labindex S.A., Warsaw, Poland.

### 2.2. Biological Material

A total of 103 strains of LAB were used for this study. These were: *Lactiplantibacillus plantarum* (n = 38), *Pediococcus pentosaceus* (n = 20), *Pediococcus acidilactici* (n = 17), *Levilactobacillus brevis* (n = 9), *Lacticaseibacillus paracasei* (n = 3), *Lacticaseibacillus rhamnosus* (n = 2), *Loigolactobacillus coryniformis* (n = 2), *Lactobacillus acidophilus* (n = 2), *Lacticaseibacillus casei* (n = 2), *Leuconostoc mesenteroides* (n = 2), *Lactobacillus delbrueckii* (n = 1), *Ligilactobacillus salivarius* (n = 1), *Pediococcus parvulus* (n = 1), *Limosilactobacillus fermentum* (n = 1), *Lentilactobacillus farraginis* (n = 1), and *Apilactobacillus kunkeei* (n = 1). LAB strains and their source of isolation (if possible) were listed in Table S1 (Supplementary file). There were 51 isolates from honeybee environment such as flowers or honey (their isolation and basic characteristics were published previously [36] and 52 collection strains (of different origins, e.g., fermented vegetables and milk products, infant feces) acquired from the own collection of the Department of Environmental Biotechnology and from the Pure Culture Collection (ŁOCK 105) of the Lodz University of Technology. *A. kunkeei* DSM 12361, which is a strain naturally inhabiting the honeybee gut, was used as a control (reference) strain.

Additionally, 21 microorganisms threatening the health of honeybees were taken to the antagonism testing. These were honeybee pathogens, opportunistic pathogens, or isolates from flowers or honeybee products: bacteria (*Paenibacillus larvae* ATCC 25367 and ATCC 49843, *Paenibacillus alvei* DSM 29, *Paenibacillus apiarius* DSM 5582, *Lysinibacillus sphaericus* DSM 1866, *Melissococcus plutonius* DSM 29964; *Bacteroides faecis* DSM 247798, *Bacteroides intestinalis* DSM 17393, *Erwinia persicina* 40, *Pantoea agglomerans* 43, *Enterobacter kobei* 40, *Enterobacter cloacae* 41); fungi (*Aspergillus niger* ATCC 16404, *Aspergillus flavus* ŁOCK CPC 0600, *Aspergillus fumigatus* ŁOCK CPC 1097) and yeast (*Aureobasidium pullulans* DSM 3042; *Zygosaccharomyces rouxii* 26D and 28D, *Candida magnoliae* 27D, *Metchnikowia pulcherrima* 40D). 

Some of the strains were purchased from the American Type Culture Collection (labeled as ATCC) or the German Collection of Microorganisms and Cell Cultures GmbH (labeled as DSM). Strains assigned as ŁOCK were acquired from ŁOCK 105 collection (as mentioned before). Strains with a numeric-letter symbol were from the own collection of the Department of Environmental Biotechnology and they were isolated from the honeybee environment (honey, flowers), with which honeybees have direct contact. *Escherichia coli* ATCC 25922 was used as a reference strain recommended for antagonism testing [20,37,38].

### 2.3. Culture, Propagation, Freezing, and Storage of Microorganisms

Microorganisms were cultured at the following media: 102 LAB strains on MRS broth and agar; *A. kunkeei* DSM 12361 on MRS broth and agar with the addition of fructose (10 g/L) and 0.05% cysteine-hydrochloride (MRS-F); *P. larvae* ATCC 25367 and ATCC 49843, *P. alvei* DSM 29, *P. apiarius* DSM 5582, *L. sphaericus* DSM 1866, *E. coli* ATCC 25922, *E. persicina* 40, *P. agglomerans* 43, *E. kobei* 40, *E. cloacae* 41 on PCA and TSB; *Z. rouxii* 26D and 26D, *C. magnoliae* 27D, *M. mulcherrima* 40D and *A. pullulans* DSM 3042 on YGC and YPG; *A. niger* ATCC 16404, *A. flavus* ŁOCK CPC 0600 and *A. fumigatus* ŁOCK CPC 1097 on MEA; *B. faecis* DSM 247798 and *B. intestinalis* DSM 17393 on BHI, while *M. plutonius* DSM 29964 on ABB medium. All microorganisms were stored in Cryobanks™ at −20 °C. Before conducting experiments, strains were activated, threefold passaged (3% inoculum) and later cultivated in the appropriate medium for 24 or 48 h in aerobic or anaerobic conditions (AnaeroGen^TM^ Atmosphere Generation Systems sachet), at 37 °C. 

### 2.4. Antagonistic Activity Testing 

#### 2.4.1. Agar Slab Method

LAB at the density of 1.8 × 10^9^ CFU/mL (6.0 according to McFarland Standard) were applied onto Petri dishes with MRS/MRS-F agar and incubated for 24 h at 37 °C. Subsequently, disks with a diameter of 10 mm were cut with a sterile cork borer in triplicate from the solid medium and placed on an appropriate agar medium containing strains of honeybee pathogens at the density of 6.0 × 10^8^ CFU/mL (2.0 according to McFarland Standard). After incubation for 24/48 h at 37 °C, zones of growth inhibition were measured and the diameter of the disc was subtracted from the result. To compare the antagonistic activity of the LAB strains against various microorganisms, the following criteria were adopted: the growth inhibition diameter above 16 mm—very strong inhibition, 11–15.9 mm—strong inhibition, 6–10.9 mm—moderate inhibition, 1–5.9 mm—weak inhibition, 0 mm—no inhibition [39,40].

The index of total antagonistic activity (IAA) was calculated as a sum of scores determined by the growth inhibition of bacteria (very strong—4 points, strong—3 points, moderate—2 points, weak—1 point, no inhibition—0 points) [39].

#### 2.4.2. Microtitration Plate Method

After analyzing the results of antimicrobial activity screening in the previous experiments, 24 LAB strains and 7 pathogens were selected for further study. The experiment was conducted for LAB metabolites (cell-free supernatants—CFSs). CFSs were prepared as follows: the liquid MRS/MRS-F medium was inoculated with an individual strain of LAB, and then incubated for 24 h at the appropriate temperature and the bacterial samples were centrifuged (10,733× *g*, 15 min). CFSs were prepared in 2 options of pH: physiological (it was from 3.81 to 4.51 depending on the strain) and neutralized (to eliminate the antagonistic activity of an acidic environment) thus the pH of the supernatants was adjusted to 7.0 ± 0.1 (with 0.1 M NaOH and HCl). Next, the supernatants were filtered using sterile syringe filters (0.22 μm) and frozen in test tubes until analysis at −20 °C. The tests were carried out in transparent 96-well polystyrene plates. The final concentrations of CFSs were as follows [%]: 12.5, 25, and 50 for physiological pH and 50 for neutralized pH. Each strain was tested in 4 replicates. The total volume of liquid in each well was 200 µL, and the final density of honeybee pathogen in each well was 6.0 × 10^8^ CFU/mL (2.0 according to McFarland Standard). Negative controls were pathogens cultured in an appropriate culture medium. Next, each well was inoculated, and the initial absorbance (0h) in a microplate reader (TriStar2 LB 942, Berthold Technologies GmbH & Co. KG, Bad Wildbad, Germany) at a wavelength of 540 nm was measured. The plates were incubated for 24 h at 37 °C, and subsequently, the absorbance was re-measured. The bacterial growth inhibition (%) was determined using Equation (1), as follows:

(1) $$\mathrm{Bacterial}\;\mathrm{growth}\;\mathrm{inhibition}\;\left(\%\right)=100-\frac{A_{24}}{A_{c}}\times 100$$

where A_24_ was an average of four replicates of absorbance values at time *t* = 24 h and A_c_ was an average of four replicates of absorbance values of negative control at time *t* = 24 h.

### 2.5. Statistical Analysis

The results for the agar slab method in Table 1 and Tables S2–S4 were presented as a mean value ± SD. Non-parametric tests were used for statistical analyses, as antipathogenic activity values of LAB did not follow a normal distribution (Shapiro–Wilk test). Differences regarding the antipathogenic activity of the analyzed LAB were tested using the Kruskal–Wallis test (KW test), followed by a multiple comparison test (MCT) to indicate significant differences between the groups. The comparison of the antagonistic activity of LAB isolated from honeybee environment vs. collection strains was performed using the non-parametric U Mann–Whitney test (UMW test). The KW and UMW tests were performed using Statistica ver. 13.1 (StatSoft, Tulsa, OK, USA). 

Results obtained for antagonistic activity of CFSs follow the normal distribution and were tested using one-way ANOVA, followed by Tukey’s multiple comparisons post hoc test (TMC tests). The one-way ANOVA and TMC test were performed using OriginPro 6.1 (Northampton, MA, USA) *p* < 0.05 was considered statistically significant. 

## 3. Results and Discussion

### 3.1. Determination of the Antagonistic Activity of LAB Using the Agar Slab Method

**Figure 1:**
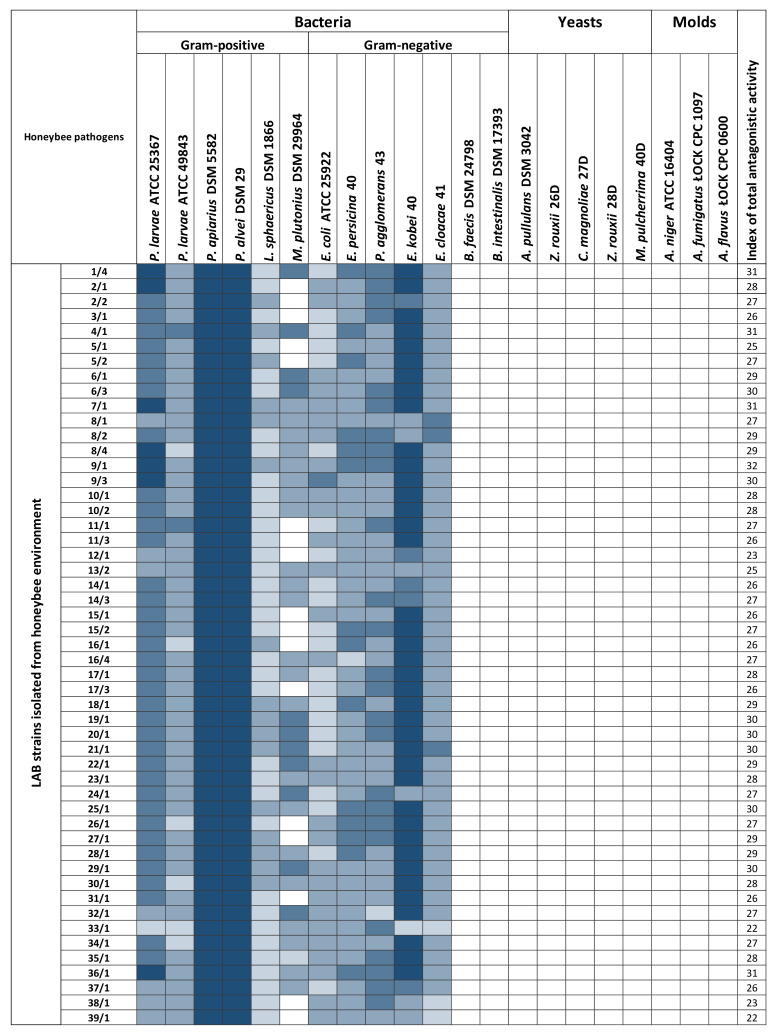

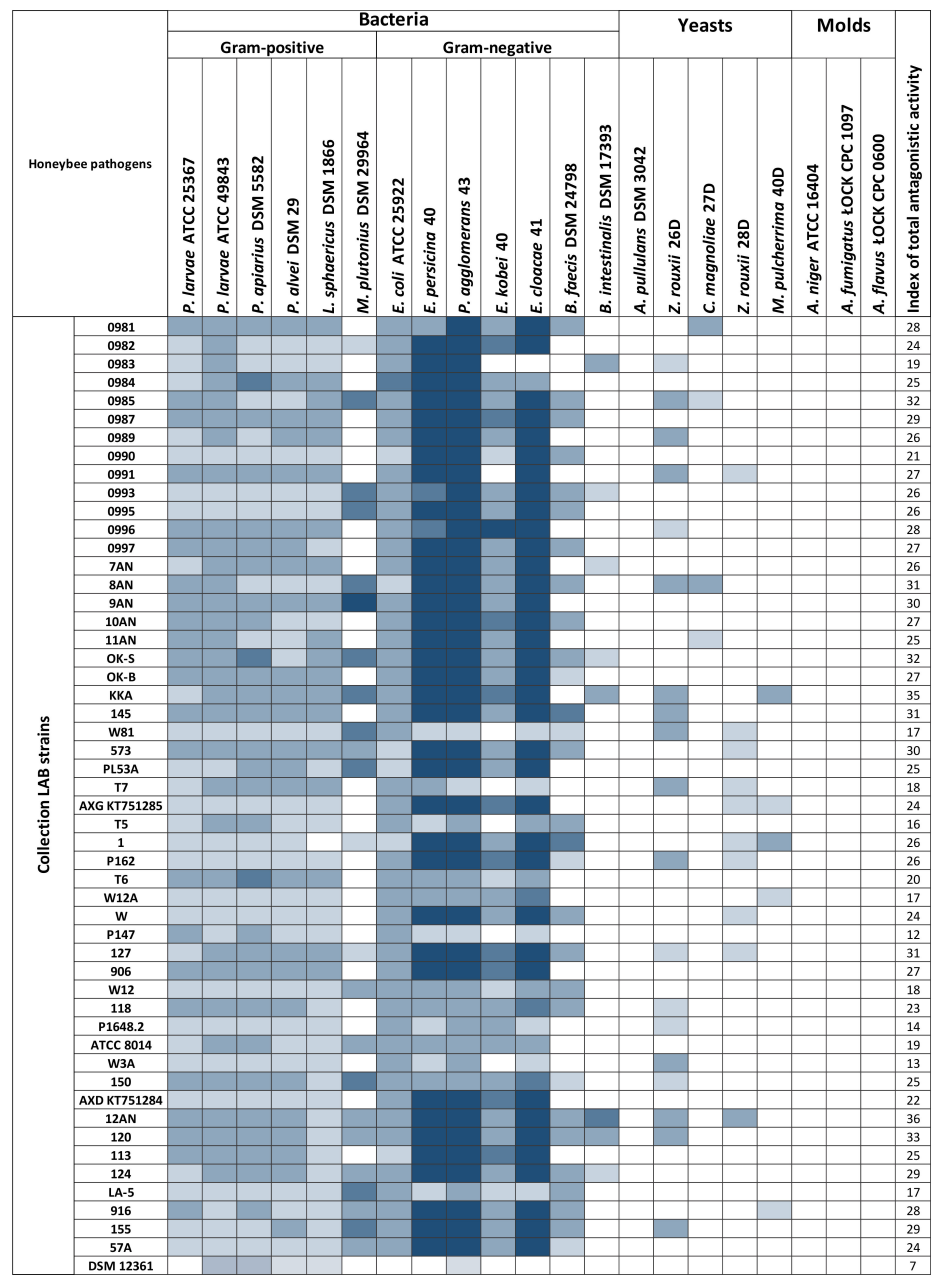
Antagonistic activity of lactic acid bacteria against honeybee pathogens and opportunistic pathogens. Type of inhibition: very dark blue—very strong, dark blue—strong, medium dark blue—moderate, light blue—weak, white—no inhibition.

**Figure 2:**
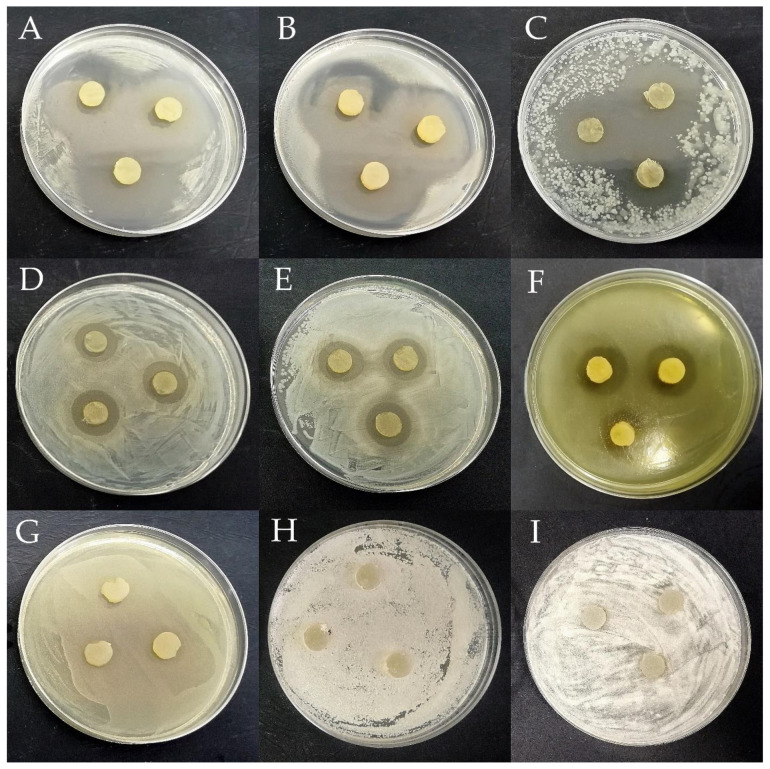
Evaluation of the antagonistic activity of lactic acid bacteria against test microorganisms: (**A**) *L. plantarum* 5/1 vs. *P. apiarius* DSM 5582; (**B**) *L. plantarum* 2/2 vs. *P. alvei* DSM 29; (**C**) *L. plantarum* 20/1 vs. *E. kobei* 40; (**D**) *P. pentosaceus* 6/3 vs. *P. larvae* ATCC 25367; (**E**) *P. acidilactici* 2/1 vs. *P. larvae* ATCC 49843; (**F**) *L. plantarum* 124 vs. *M. plutonius* DSM 29964; (**G**) *L. plantarum* 5/1 vs. *B. faecis* DSM 247798; (**H**) *P. acidilactici* 4/1 vs. *M. pulcherrima* 40D, and (**I**) *P. pentosaceus* 34/1 vs. *A. fumigatus* ŁOCK CPC 1097.

Antagonistic activity is a crucial factor in evaluating the probiotic properties of LAB. Due to their widespread occurrence, LAB exhibit a broad and varied spectrum of combating pathogenic microorganisms and have the potential to biocontrol diseases of honeybees caused by bacteria and fungi [30,41,42,43]. The antagonistic activity of 103 LAB strains was tested against 21 honeybee pathogens (Figure 1). All tested LAB strains displayed the ability to inhibit the growth of some pathogens/opportunistic pathogens. Examples of growth inhibition zones are shown in Figure 2. Each LAB strain exhibited a unique spectrum of antagonistic activity, and the inhibition of pathogen growth varied depending on the strain evaluated. Detailed data with values, standard deviations, and statistical differences regarding the antipathogenic activity of the analyzed LAB are presented in Tables S2 and S3 included in Supplementary Materials. 102 out of all 103 LAB strains tested inhibited to varying degrees the growth of bacteria of the genus *Paenibacillus*. All 103 LAB strains inhibited the growth of *P. larvae* ATCC 49843, *P. apiarius* DSM 5582, and *P. alvei* DSM 29, which are well-known honeybee pathogens [8,44,45]. Frequent growth inhibition was also noted in the case of *E. persicina* 40, *P. agglomerans* 43, and *L. sphaericus* DSM 1866, where 102 out of 103 LAB strains (99.03% coverage) displayed antagonistic activity. Molds and yeasts turned out to be the most resistant among the tested microorganisms. In the case of *A. pullulans* DSM 3402, *Z. rouxii* 26D and 28D, *C. magnoliae* 27D, and *M. pulcherrima* 40D, the number of LAB strains displaying antagonistic activity was 0 (0% coverage), 18 (17.7% coverage), 10 (9.7% coverage), 4 (3.9% coverage), and 5 (4.9% coverage). After screening the results, the molds turned out to be fully resistant to the activity of all tested LAB strains, and no zones of inhibition were observed. Isolates from the honeybee environment showed a statistically increased inhibitory effect on the common honeybee pathogens: *P. larvae* ATCC 25367 and ATCC 49843, *M. plutonius* DSM 29964, *P. apiarius* DSM 5582, and *P. alvei* DSM 29 (*p* ≤ 0.05). On the other hand, collection LAB strains showed more potent antimicrobial activity against the *L. sphaericus* DSM 1866, *E.coli* ATCC 25922, *E. persicina* 40, *P. agglomerans* 43, and *E. cloacae* 41 (*p* ≤ 0.05). A comparative statistical analysis of the antagonistic activity of LAB strains by origin source is presented in Table 1. Of the 103 tested LAB strains, 55 showed antagonistic activity against *M. plutonius* DSM 29964; however, very strong inhibition was noted only in the case of *L. salivarius* 9AN, where the growth inhibition diameter reached 18.00 mm ± 1.00 mm (Table S2). Growth of *P. larvae* ATCC 25367 was very strongly inhibited by *P. acidilactici* 1/4, 2/1, 9/1, and 36/1, *P. pentosaceus* 9/3, and *L. plantarum* 8/4 and 17/1. Comparing the two tested *P. larvae* strains, the growth of *P. larvae* ATCC 49843 was less inhibited, suggesting that LAB antagonistic activity also depends on the pathogen strain of the same species. 

Additionally, LAB strains isolated from the honeybee environment showed more potent growth inhibition of Gram-positive bacteria, and the collection LAB strains exhibited stronger antagonism against Gram-negative bacteria (Figure 1). LAB strains were also examined in terms of division into species, which shows that *L. plantarum* inhibits the growth of the broadest spectrum of microorganisms (Table 2). For the remaining LAB, more strains belonging to a given species should be tested to obtain more reliable results. The list of LAB with statistically significant antagonistic activity against bacteria is presented in Table S4 included in Supplementary Materials.

The highest index of total antagonistic activity (IAA) was demonstrated by *L. casei* 12AN (IAA = 36) and *L. brevis* KKA (IAA = 35), which inhibited the growth of most of 21 pathogens/opportunistic pathogens of honeybees, including yeast. The lowest IAA and weak ability to inhibit the growth of microorganisms was demonstrated by the *A. kunkeei* DSM 12361 (IAA = 7), which displayed antagonism towards *P. larvae* ATCC 25367 and ATCC 49843, *P. alvei* DSM 29, *P. apiarius* DSM 5582 and *P. agglomerans* 43. 

LAB antagonistic activity is a widely studied topic both in veterinary medicine and agriculture due to their antimicrobial activity [46,47]. Honeybee pathogens pose a significant threat to beekeeping and the environment; thus finding a natural way to combat or inhibit their growth is an important issue [4]. One of the best-known and studied honeybee pathogens is *P. larvae*, the causing agent of American foulbrood disease. Forsgren et al. in their study showed that *A. kunkeei* CCUG 53901 has only a selective and partial antagonistic activity against *P. larvae* LMG 16247 and the ability to inhibit the growth of this pathogen depended on the synergistic action of factors such as the production of antibacterial peptides, hydrogen peroxide, bacteriocins, and metabolic end products [48]. Individual LAB phylotypes affected the growth of pathogens in various degrees, due to their unique properties and differences in fermentation end products [48]. In our study, *A. kunkeei* DSM 12361 was the reference strain as a species commonly found in honeybee products and naturally inhabiting the digestive tract of honeybees [49]. According to Iorizzo et al., different strains of *A. kunkeei* exhibit various levels of antagonistic activity, the highest for cell lysates and culture broth, which also applies to other LAB strains [50]. The results presented in this article suggest that *A. kunkeei* DSM 12361 displayed lower antagonistic activity against the tested microorganisms than the remaining 102 LAB strains, but it was against key pathogens such as *P. larvae*. According to in vivo tests conducted by Lindenfelser, the growth of *P. larvae* was inhibited by the use of propolis, which is a rich source of LAB [51]. Yoshiyama et al. in their study demonstrated the strong inhibitory ability of LAB towards *P. larvae* RIAS No. P1 GIFU-1 strain and suggested a relationship between the antagonism and the mechanisms related to the LAB antibacterial activity (e.g., production of lactic acid inhibiting bacterial growth by lowering pH) [52]. Due to the pathogenic effect of *P. larvae* on honeybees, the inhibition of the growth of this microorganism is a desirable ability to select appropriate LAB strains to construct a preparation intended to protect these insects. *P. alvei* and *P. apiarius* also negatively affect the viability of honeybee colonies. *P. alvei* is a secondary invader for European Foulbrood disease caused by *M. plutonius* [7]. This saprophytic bacterium is found in chronically diseased colonies, growing in larval remains. *P. apiarius* is considered a threat to honeybees, but this issue has not yet been thoroughly investigated [8]. *P. larvae*, *P. alvei*, and *P. apiarius* are spore-forming Gram-positive rods, aerobic or facultatively anaerobic. Despite the similarities between species belonging to the same genus, strains of pathogens show different resistance to the antagonistic activity of other microorganisms [53]. According to Keller et al., there is a separation between *P. larvae*, *P. apiarius*, and *P. alvei* with a comparatively distant phylotypic topology relationship between them [8]. In a phylogenomic tree, *P. alvei* and *P. apiarius* are clustered into one group, distinguished from *P. larvae* [8]. In our study, all tested LAB strains demonstrated antagonistic activity against bacillus bacteria. The largest zones of growth inhibition were observed for *P. apiarius* DSM 5582, and the smallest for *P. larvae* ATCC 25367. *L. sphaericus* is another known pathogenic bacterium causing lethal diseases in honeybee broods. Infected colonies often do not have a queen and the worker honeybee population is reduced. Dead larvae resemble a ropy mass similar to larvae remains found in colonies infected by *P. larvae* [54]. The tested LAB strains displayed moderate antimicrobial activity against *L. sphaericus* DSM 1866, where the largest diameter of growth inhibition reached 9.67 mm ± 0.58 mm for *L. mesenteroides* T7. Pietropaoli et al. demonstrated moderate LAB antagonism against *M. plutonius* and a reduction in the insurgence of cases of European foulbrood [55]. The results presented by the authors of this article showed that LAB strains isolated from the honeybee environment more often inhibited the growth of this pathogen as compared to collection strains (Table 1); however, zones of inhibition in the case of collection LAB strains were larger (Tables S2 and S3). *E. coli* ATCC 25922 was used for the antagonism study presented above as the reference strain and as an opportunistic pathogen of honeybees [56]. Chang et al. suggested a negative impact of these bacteria on honeybee health by reducing colony lifespan, increasing gut permeability, and impairing learning ability [57]. The moderate inhibition of *E. coli* ATCC 25922 growth may be explained by the sensitivity of Gram-negative bacteria to organic acids such as lactic acid, which is the end product of the lactic fermentation by LAB [58]. 

The research also investigated bacteria that may be potential opportunistic pathogens of honeybees. *P. agglomerans*, *E. cloacae*, *E. kobei*, and *E. persicina* belong to the natural microbiota of flowers, are detectable in the digestive tract of honeybees, and are known human opportunistic pathogens [59,60,61,62,63]. Currently, no research has been undertaken whether these bacteria may be opportunistic pathogens of honeybees, e.g., when honeybees are weakened by pesticides or antibiotics or when their immunity is lowered due to infection. The tested LAB strains showed strong antagonistic activity against these bacteria. The largest diameter of the growth inhibition zone (35.67 mm ± 1.53 mm) was recorded for *L. brevis* KKA against *E. cloacae* 41. *B. intestinalis* and *B. faecis* are bacteria isolated from human feces and are known opportunistic pathogens [64]. There are currently no studies on the potential effects of these bacteria on honeybees. Growth inhibition was more common in the case of *B. faecis* DSM 24789 and only in the case of LAB isolated from the honeybee environment.

Subsequently, studies were conducted on various species of fungi that threaten honeybees. Several studies have attempted to characterize honeybee-associated yeast communities [11,65,66,67,68]. *Z. rouxii* and *M. pulcherrima* are commonly detectable in honeybee products, and *C. magnoliae* was isolated directly from *A. mellifera* L. [65,66,67,68]. *M. pulcherrima* is also a rare opportunistic pathogen in humans, responsible for onychomycosis, root caries lesions, respiratory diseases, and a few cases of bloodstream infections [69,70]. However, the impact of these yeasts on honeybee health has not yet been thoroughly investigated. Another microorganism tested was *A. pullulans,* which is a pathogenic yeast-like fungus that infects scaly insects and causes melanosis in honeybees [11]. Regarding mold, it was decided to investigate species belonging to the *Aspergillus* genus. These pathogenic molds cause stonebrood disease in honeybees. The symptoms of stonebrood are hard mummified larvae found in brood cells and, less commonly, infected adults [71]. Inhibition of fungal growth depends individually on the pathogen tested and the LAB strain, thus antagonistic activity is a strain-dependent property also in the case of fungi [72]. In the study presented above, only a few LAB strains displayed antagonistic activity towards yeasts, in the case of mold, growth inhibition was not observed. The largest diameter of the growth inhibition zone (12.67 mm ± 2.08 mm) was recorded for the strain *L. plantarum* 8AN against *Z. rouxii* 26D.

The results indicate a stronger growth inhibitory ability of LAB strains isolated from the honeybee environment and collection LAB strains compared to *A. kunkeei*, a strain that naturally inhabits the honeybee gut. It may suggest the need to select LAB strains with the strongest antagonistic (probiotic) properties to strengthen the resistance of honeybees against pathogens.

### 3.2. Antagonistic Activity of LAB Metabolites 

**Figure 3:**
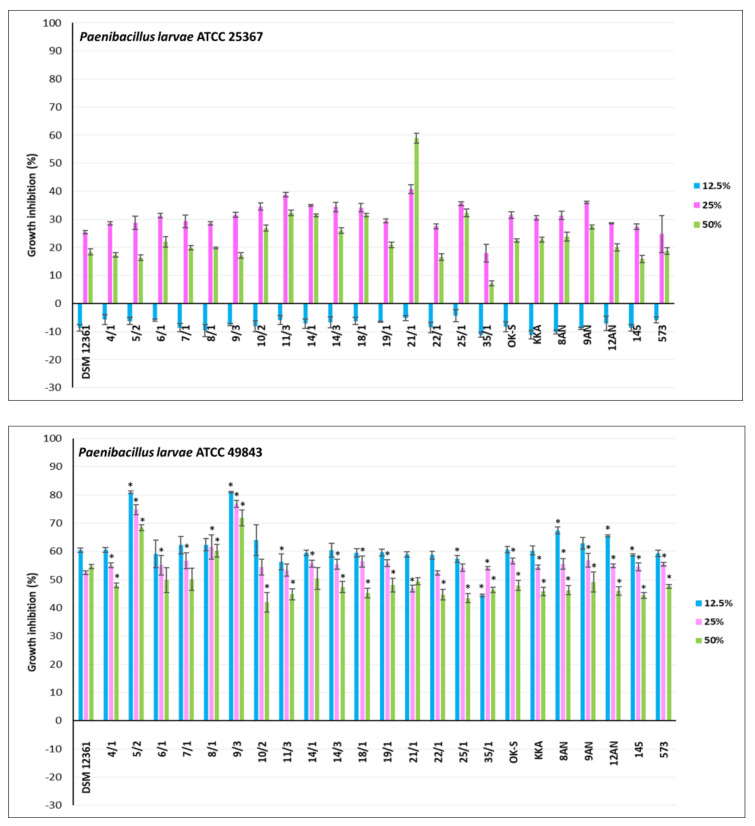

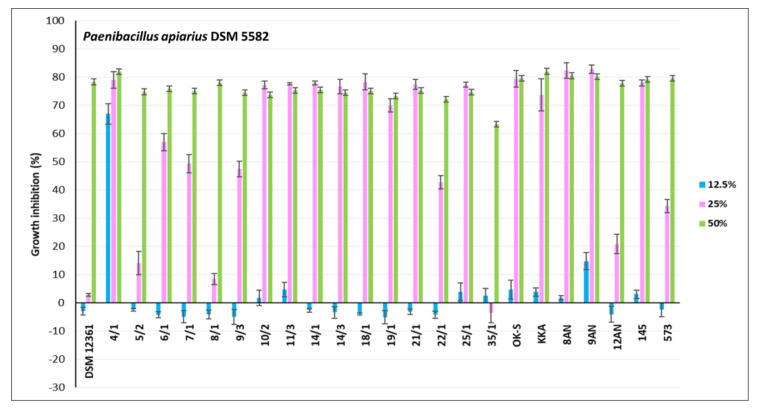
Growth inhibition (%) of *P. larvae* ATCC 25367, *P. larvae* ATCC 49843, and *P. apiarius* DSM 5582 by cell−free supernatants (culture metabolites, at physiological pH) of lactic acid bacteria evaluated by microtitration method. Each data point represents the mean from four individual wells. Results are presented as mean ± standard deviation (SD). * Statistically significant difference in growth inhibition compared to *A. kunkeei* DSM 12361 at an equivalent concentration at *p* ≤ 0.05.

**Figure 4:**
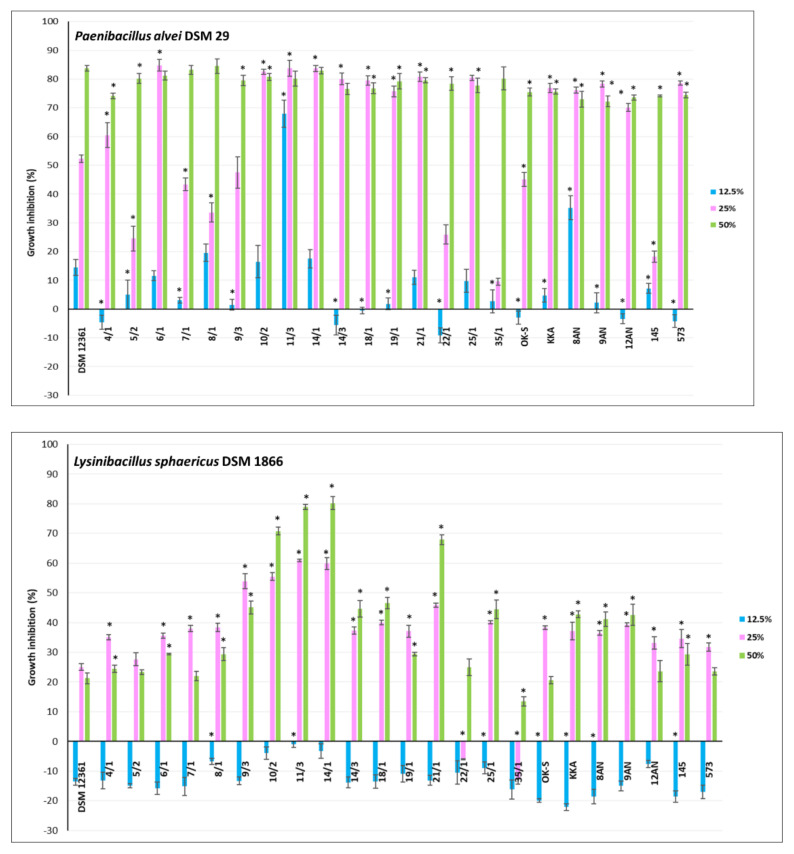

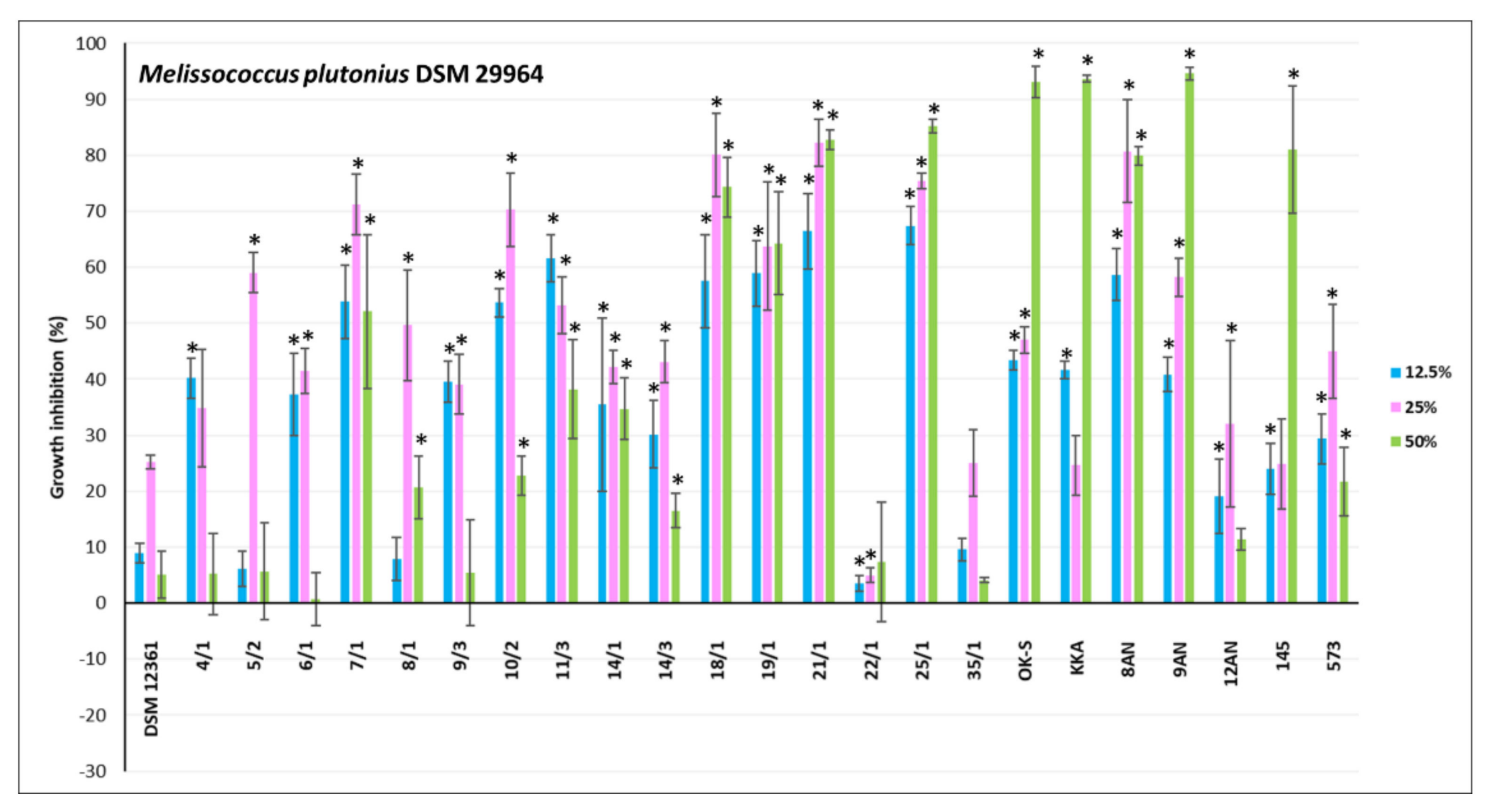
Growth inhibition (%) of *P. alvei* DSM 29, *L. sphaericus* DSM 1866 and *M. plutonius* DSM 29964 by cell−free supernatants (culture metabolites, at physiological pH) of lactic acid bacteria evaluated by microtitration method. Each data point represents the mean from four individual wells. Results are presented as mean ± standard deviation (SD). * Statistically significant difference in growth inhibition compared to *A. kunkeei* DSM 12361 at an equivalent concentration at *p* ≤ 0.05.

**Figure 5:**
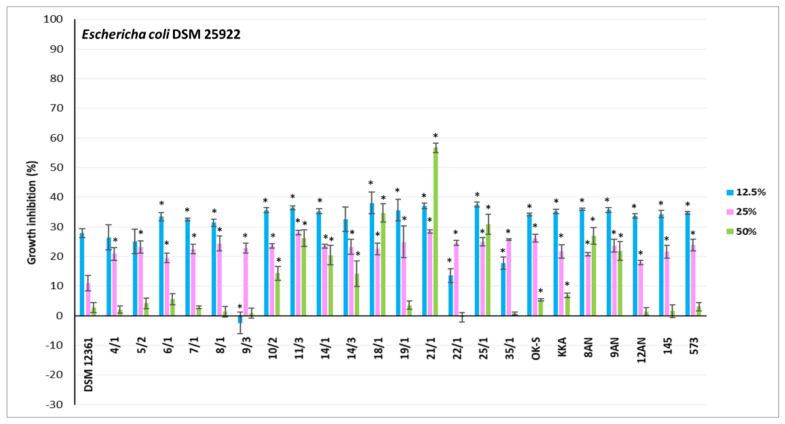
Growth inhibition (%) of *E. coli* ATCC 25922 by cell−free supernatants (culture metabolites, at physiological pH) of lactic acid bacteria evaluated by microtitration method. Each data point represents the mean from four individual wells. Results are presented as mean ± standard deviation (SD). * Statistically significant difference in growth inhibition compared to *A. kunkeei* DSM 12361 at an equivalent concentration at *p* ≤ 0.05.

CFS refers to a liquid medium obtained by filtration of a grown bacterial culture, containing organic acids and secondary metabolites of bacterial growth [73]. Previous studies have shown the antimicrobial activity of CFSs against fungi and bacteria [74,75,76]. The antagonistic activity of LAB is mainly due to lactic and acetic acids, products of central carbon metabolism [29]. CFSs from LAB also may contain other antimicrobial metabolites such as reuterin, hydrogen peroxide, bacteriocins, and products of peptide synthesis and bioconversion [77]. The CFSs content varies depending on the genus and species of LAB [78]. In our study, CFSs displayed different levels of antimicrobial activity against honeybee pathogens. For the experiment, 23 LAB showing the strongest antagonism in the agar slab method were selected. The results for inhibition of pathogen growth by CFSs from LAB are shown in Figure 3, Figure 4 and Figure 5. Moreover, the selection of the above strains was guided also by the fact that they should be of different genera and species. Their antagonistic properties, especially against *Paenibacillus* strains, have also been considered. Therefore, the following LAB strains were selected for further research: *P. acidilactici* 4/1, 5/2, 6/1, 7/1, 8/1, 22/1, 25/1, 35/1, *L. plantarum* 10/2, 14/3, 18/1, 21/1, 8AN, 145, *P. pentosaceus* 9/3, 11/3, 14/1, 19/1, *P. parvulus* OK-S, *L. brevis* KKA, *L. salivarius* 9AN, *L. casei* 12AN, and *L. acidophilus* 573. *A. kunkeei* DSM 12361 was selected as the control. Most LAB strains showed statistically significant growth inhibition of the pathogens tested; however, the results differed depending on the concentrations used (*p* ≤ 0.05) (Figure 3, Figure 4 and Figure 5). The highest antagonistic activity against *P. larvae* ATCC 25367 was demonstrated by CFS from *L. plantarum* 21/1, where the growth inhibition of this pathogen reached 59.92% ± 1.80% at the concentration of 50% of the CFS (Figure 3). Results of MODA assay conducted by Babrud et al. showed a strong antimicrobial activity of LAB metabolites against *P. larvae* and inhibition of biofilm production due to low pH [79]. CFS from *L. reuteri* ATCC 23272 displayed inhibitory effects on *P. larvae* KB10 growth and the diameter of the growth inhibition was 12.75 ± 3.2 mm at 1000 µL/mL for minimum bactericidal concentrations (MBC) and minimum inhibitory concentrations (MIC) [79]. The agar-diffusion assay performed by Audisio et al. showed antagonistic activity of CFS from *Lactobacillus johnsonii* AJ5, IG9 and CRL1647 against all tested *P. larvae* strains (i.e., I, II, III, IV, Azul, C, 7 and 35), suggesting strong antagonism of LAB metabolites against this pathogen [80]. In the current research, in most test pathogens, the greatest inhibition of their growth was observed in the presence of CFSs concentrations of 25 and 50%. For most CFSs tested, the inhibition of *P. larvae* ATCC 25367 growth was higher for the concentration of 25% of LAB metabolites. Some LAB produce heat-sensitive metabolites such as ethanol and carbon dioxide. Evaporation of these compounds can stimulate the growth of *P. larvae* [81,82]. The strong antagonism demonstrated by *L. plantarum* 21/1 metabolites against *P. larvae* ATCC 25367 at 50% concentration may indicate a high antibacterial activity of metabolites of this strain even under conditions favorable to the development of this pathogen. The weakest antagonism against *P. larvae* ATCC 25367 was noted for CFS from *P. acidilactici* 35/1 (Figure 3). The growth inhibition was up to 17.96% ± 3.17%. The results for all tested CFSs from LAB for a sample concentration of 12.5% demonstrated stimulation of *P. larvae* ATCC 25367 growth. A similar conclusion can be drawn after analyzing the results obtained for inhibiting the growth of *L. sphaericus* DSM 1866 (Figure 4). The low antagonistic activity of the samples at a concentration of 12.5% may be due to the low concentration of antimicrobial metabolites. That is why it is so important to determine a concentration of bacteria in probiotic preparations to show the strongest activity, which must be evaluated under experimental conditions in vivo. Moreover, growth inhibition of *P. larvae* ATCC 49843 (Figure 3) significantly differed from the results obtained for *P. larvae* ATCC 25367, even though these strains belonged to the same species. The tested CFSs from LAB strongly inhibited the growth of *P. larvae* ATCC 49843 at all tested concentrations (Figure 3). The differences in antimicrobial activity did not differ significantly with the concentrations of CFSs used. The inhibition of *P. larvae* ATCC 49843 growth was the strongest for CFSs from *P. pentosaceus* 9/3 and reached 80.99% ± 0.26% for the concentration of 12.5%. Antimicrobial activity is strain-dependent, and CFSs exhibited a broad spectrum of antagonism against the pathogens tested. According to Iorizzo et al., the antagonistic activity of LAB metabolites against honeybee pathogens differs depending on the test strain [83]. After performing agar well diffusion assay, CFS from *L. plantarum* strains (P8, P25, P86, P95, and P100) displayed differential antagonism against *P. larvae* ATCC 9545 and growth inhibition diameters ranged from 3.4 ± 0.1 to 5.8 ± 0.3 mm [82]. In the current study, all tested CFSs strongly inhibited the growth of *P. apiarius* DSM 5582 and *P. alvei* DSM 29, where the strongest antimicrobial properties were demonstrated by *P. acidilactici* 4/1 and *P. pentosaceus* 11/3, respectively (Figure 3 and Figure 4). CFS from *P. pentosaceus* 11/3 showed strong antagonism against *P. alvei* DSM 29 even for a 12.5% concentration, demonstrating extensive antibacterial properties against this pathogen. The growth of *M. plutonius* DSM 29964 was most severely inhibited by CFSs from *L. salivarius* 9AN, *P. parvulus* OK-S, and *L. brevis* KKA (Figure 4). CFS from *L. plantarum* 14/1 showed the highest antibacterial activity against *L. sphaericus* DSM 1866, where the growth inhibition reached 80.22% ± 2.18% for the 50% concentration. The weakest antagonism was observed in the case of *E. coli* ATCC 25922 (Figure 5). CFSs from *L. plantarum* 21/1 displayed the strongest antagonistic activity, where the growth inhibition reached 56.64% ± 1.63% for the 50% concentration. Several studies were undertaken to determine the antagonistic activity of LAB metabolites against *E. coli*. According to Portella et al., CFSs inhibited the growth of *E. coli* in vitro by 92% [84]. The low pH, organic acids, and possibly bacteriocin-like substances influenced the antibacterial activity of the metabolites [84]. According to Bian et al., CFS from *L. reuteri* DPC16 grown in MRS broth supplemented with glycerol (MRSg) showed strong, pH-independent, dose-dependent antagonism that affected both Gram-negative and Gram-positive pathogens [85]. The presence of reuterin in CFSs from LAB strains grown in MRSg broth enhanced antagonism against *E. coli* and increased inhibition of the growth of this pathogen by up to 100% [85]. According to the authors’ knowledge, there are no in vivo tests raising the issue of the antimicrobial activity of LAB metabolites on honeybee pathogens. The results obtained for the reference strain, *A. kunkeei* DSM 12361, displayed the weakest antibacterial activity of the metabolites of this strain against all pathogens tested (*p* ≤ 0.05). In addition, CFSs from LAB isolated from the honeybee environment showed stronger antagonism against *L. sphaericus* DSM 1866 and *P. larvae* ATCC 49843; however, in the case of *M. plutonius* DSM 29964, stronger growth inhibition was exhibited by CFSs from collection LAB strains. In the case of the other pathogens, the origin did not matter.

The antagonistic activity of the CFSs from LAB at neutralized pH was tested for example three strains—*P. larvae* ATCC 25367, *P. apiarius* DSM 5582, and *L. sphaericus* DSM 1866 (Table 3). The strongest antagonism was displayed by strain *P. acidilactici* 35/1 against *L. sphaericus* DSM 1866 (36.25% ± 12.74%), and the weakest by *P. acidilactici* 5/2 against *P. apiarius* DSM 5582 (3.10% ± 1.31%). All LAB strains except *L. plantarum* 14/3 showed statistically significant growth inhibition of *L. sphaericus* DSM 1866 and *P. larvae* ATCC 25367 (*p* ≤ 0.05) towards *A. kunkeei* DSM 12361. Despite the neutralized pH, other LAB metabolites such as bacteriocins, sakacin T-α, sakacin T-β, N-acetylmuramidase, and H_2_O_2_ may demonstrate strong antibacterial activity [74,86]. Dysbiosis in the intestinal microbiota of honeybees may lead to a neutralization of the pH, making them susceptible to infections by neutrophiles, such as *Paenibacillus* spp. or *E. coli*. Generally, CFSs at neutralized pH showed weaker antibacterial activity compared to CFSs at physiological pH. This confirms the theory that the basic mechanism of LAB antagonistic activity is the acidification of the environment. Currently, there are no in vivo and in vitro tests on the antagonism of CFSs at neutralized pH. The study on the effect of pH on antimicrobial activity was undertaken by Audiosio et al. [80]. After adjusting the pH of CFS to 6.0, the metabolites of all tested LAB strains displayed no antagonistic activity, suggesting a significant effect of acids in inhibiting the growth of various pathogens [80]. The above studies demonstrate the antibacterial activity of LAB metabolites at physiological and neutralized pH. It is, therefore, necessary to conduct in vivo tests in the future and determine the role of CFSs in the maintenance of the viability of honeybees and the influence of metabolites on the prophylactic strategy against diseases caused by honeybee pathogens.

## 4. Conclusions

While honeybees’ environmental and economic impact is well known, there is a growing need to find an ecological way to combat the pathogenic microorganisms that threaten these insects. By evaluating the effects of LAB on the growth of honeybee pathogens, this study established that all tested LAB strains exhibited various levels of antagonism. The antimicrobial activity of LAB is unique against microorganisms belonging to the same species. LAB strains isolated from the honeybee environment demonstrated more potent growth inhibition of known honeybee pathogens, such as *Paenibacillus* species. However, the collection LAB strains exhibited stronger antagonistic activity against opportunistic pathogens. Among the examined microorganisms, molds and yeasts turned out to be the most resistant to the antagonistic action of LAB strains. The strongest antimicrobial activity was displayed by cocci represented by the genus *Pediococcus* and the species *P. acidilactici* and *P. pentosaceus.* Additionally, strong antagonistic activity demonstrated also bacilli of the species *L. plantarum*. All bacteria mentioned above were mostly isolated from the honeybee environment (flowers, honey). The tested LAB strains exhibited stronger antagonism compared to the natural symbiont of honeybees, i.e., *A. kunkeei* DSM 12361, suggesting the need to select these bacteria to strengthen honeybees’ immune systems. Additionally, the results obtained after testing the antagonistic activity of LAB metabolites showed various levels of growth inhibition depending on the pathogen tested, because each CFS demonstrated a unique spectrum of antimicrobial activity. After comparing the results obtained for CFSs at physiological and neutralized pH, the theory of acidification of the environment as the basic mechanism of LAB antimicrobial activity was confirmed. The use of a LAB-based strategy in the biocontrol and prevention of pathogenic honeybee microbes offers interesting perspectives. As a result of the conducted research, LAB strains that exhibited the strongest antagonism against the tested pathogenic microorganisms will be selected for future in vitro tests, such as adherence abilities to biotic and abiotic surfaces, pesticide detoxification, antibiotic resistance, survival in sugar syrup, or the simulated gastrointestinal conditions. The results of this study may in the future contribute to the selection of LAB strains with the best probiotic properties for the construction of an ecological preparation to improve the viability of honeybees. 

## Figures and Tables

**Table 1:** Analysis of the antagonistic activity of lactic acid bacteria (LAB) isolated from honeybee environment compared to collection LAB strains. The results were obtained by comparing the mean diameters of growth inhibition (mm).

	Isolates from Honeybee Environment	Collection Strains	*p*-Value (U Mann–Whitney Test)	
*P. larvae* ATCC 25367	16.92 ± 2.92	5.74 ± 1.85	0.0000	ENV *
*P. larvae* ATCC 49843	7.82 ± 1.74	6.29 ± 1.39	0.0000	ENV
*P. apiaries* DSM 5582	31.83 ± 4.84	6.42 ± 2.15	0.0000	ENV
*P. alvei* DSM 29	29.45 ± 4.47	5.74 ± 1.29	0.0000	ENV
*L. sphaericus* DSM 1866	4.72 ± 1.89	5.55 ± 1.88	0.0001	
*M. plutonius* DSM 29964	6.57 ± 5.09	4.89 ± 5.91	0.0236	ENV
*E. coli* ATCC 25922	6.46 ± 2.13	7.73 ± 1.94	0.0000	
*E. persicina* 40	9.54 ± 2.28	15.55 ± 6.89	0.0000	
*P. agglomerans* 43	10.44 ± 2.61	18.12 ± 7.06	0.0000	
*E. kobei* 40	19.22 ± 6.29	7.69 ± 4.31	0.0000	ENV
*E. cloacae* 41	8.28 ± 2.52	20.05 ± 10.23	0.0000	
*B. faecis* DSM 24798	0.00	4.65 ± 4.51	0.0000	
*B. intestinalis* DSM 17393	0.00	0.92 ± 2.35	0.0000	
*A. pullulans* DSM 3042	0.00	0.00	*p* > 0.05	
*Z. rouxii* 26D	0.00	2.75 ± 4.20	0.0000	
*C. magnoliae* 27D	0.00	0.33 ± 1.68	0.0127	
*Z. rouxii* 28D	0.00	0.67 ± 1.99	0.0000	
*M. pulcherrima* 40D	0.00	0.35 ± 1.57	0.0007	
*A. niger* ATCC 16404	0.00	0.00	*p* > 0.05	
*A. fumigatus* ŁOCK CPC 1097	0.00	0.00	*p* > 0.05	
*A. flavus* ŁOCK CPC 0600	0.00	0.00	*p* > 0.05	

* ENV—pathogens that were statistically stronger inhibited by isolates from the honeybee environment than by the collection strains.

**Table 2:** The number of active lactic acid bacteria species against honeybee pathogens and opportunistic pathogens.

LAB Strains (n = 103)	Bacteria													Yeasts					Molds		
	Gram-Positive						Gram-Negative														
	*P. larvae* ATCC 25367	*P. larvae* ATCC 49843	*P. apiaries* DSM 5582	*P. alvei* DSM 29	*L. sphaericus* DSM 1866	*M. plutonius* DSM 29964	*E. coli* ATCC 25922	*E. persicina* 40	*P. agglomerans* 43	*E. kobei* 40	*E. cloacae* 41	*B. faecis* DSM 24798	*B. intestinalis* DSM 17393	*A. pullulans* DSM 3042	*Z. rouxii* 26D	*C. magnoliae* 27D	*Z. rouxii* 28D	*M. pulcherrima* 40D	*A. niger* ATCC 16404	*A. fumigatus* ŁOCK CPC 1097	*A. flavus* ŁOCK CPC 0600
*P. acidilactici* (n = 17)	17	17	17	17	17	14	17	17	17	17	17	0	0	0	0	0	0	0	0	0	0
*L. plantarum* (n = 38)	38	38	38	38	38	19	38	38	38	37	38	14	3	0	10	2	4	2	0	0	0
*P. pentosaceus* (n = 20)	20	20	20	20	20	11	20	20	20	20	20	0	0	0	0	0	0	0	0	0	0
*L. brevis* (n = 9)	9	9	9	9	8	3	9	9	9	6	8	3	7	0	6	0	3	2	0	0	0
*L. paracasei* (n = 3)	3	3	3	3	3	3	3	3	3	3	3	3	1	0	1	1	0	1	0	0	0
*L. rhamnosus* (n = 2)	2	2	2	2	2	1	2	2	2	2	2	1	0	0	0	0	0	0	0	0	0
*L. coryniformis* (n = 2)	2	2	2	2	2	0	2	2	2	2	2	1	0	0	0	1	0	0	0	0	0
*L. acidophilus* (n = 2)	2	2	2	2	2	2	2	2	2	2	2	2	0	0	0	0	1	0	0	0	0
*L. casei* (n = 2)	2	2	2	2	2	1	2	2	2	2	2	1	1	0	1	0	1	0	0	0	0
*L. mesenteroides* (n = 2)	2	2	2	2	2	0	2	2	2	0	2	1	0	0	1	0	1	0	0	0	0
*L. delbrueckii* (n = 1)	1	1	1	1	1	0	1	1	1	1	1	1	0	0	0	0	0	0	0	0	0
*L. salivarius* (n = 1)	1	1	1	1	1	1	1	1	1	1	1	0	0	0	0	0	0	0	0	0	0
*P. parvulus* (n = 1)	1	1	1	1	1	1	1	1	1	1	1	1	1	0	0	0	0	0	0	0	0
*L. fermentum* (n = 1)	1	1	1	1	1	1	1	1	1	1	1	1	0	0	0	0	0	0	0	0	0
*L. farraginis* (n = 1)	1	1	1	1	1	0	1	1	1	1	1	0	0	0	0	0	0	0	0	0	0
*A. kunkeei* (n = 1)	0	1	1	1	1	0	0	0	1	0	0	0	0	0	0	0	0	0	0	0	0

n—number of strains tested within the species.

**Table 3:** Growth inhibition (%) of honeybee pathogens displayed by CFSs from lactic acid bacteria strains at pH adjusted to 7.0 ± 0.1 evaluated by microtitration method. Each data point represents the mean from four individual wells. Results are presented as mean ± standard deviation (SD). * Statistically significant difference in growth inhibition compared *A. kunkeei* DSM 12361 at *p* ≤ 0.05.

LAB Strains	*P. larvae* ATCC 25367	*P. apiarius* DSM 5582	*L. sphaericus* DSM 1866
*A. kunkeei* DSM 12361	9.22 ± 5.72	15.86 ± 6.75	3.20 ± 1.50
*P. acidilactici* 4/1	22.31 ± 1.06 *	5.39 ± 1.35 *	22.28 ± 2.58 *
*P. acidilactici* 5/2	21.58 ± 4.21 *	3.10 ± 1.31 *	26.26 ± 1.55 *
*P. acidilactici* 6/1	23.84 ± 2.59 *	12.97 ± 0.68	20.12 ± 1.95 *
*P. acidilactici* 7/1	24.73 ± 0.99 *	9.72 ± 0.34	27.89 ± 3.37 *
*P. acidilactici* 8/1	26.96 ± 1.70 *	5.18 ± 1.27 *	33.91 ± 10.17 *
*P. pentosaceus* 9/3	21.14 ± 3.24 *	8.03 ± 0.45	18.92 ± 1.37 *
*L. plantarum* 10/2	24.57 ± 1.66 *	7.99 ± 1.39	24.57 ± 1.26 *
*P. pentosaceus* 11/3	26.18 ± 1.14 *	9.24 ± 1.30	24.29 ± 1.09 *
*P. pentosaceus* 14/1	24.94 ± 0.65 *	7.61 ± 0.49	27.71 ± 2.17 *
*L. plantarum* 14/3	16.23 ± 2.40	4.78 ± 1.24 *	13.29 ± 3.23 *
*L. plantarum* 18/1	19.08 ± 3.46 *	10.05 ± 1.58	19.48 ± 1.78 *
*P. pentosaceus* 19/1	19.76 ± 3.65 *	10.28 ± 0.90	19.69 ± 1.87 *
*L. plantarum* 21/1	27.49 ± 4.53 *	15.60 ± 7.47	25.65 ± 3.01 *
*P. acidilactici* 22/1	25.86 ± 3.16 *	13.09 ± 10.25	34.99 ± 0.93 *
*P. acidilactici* 25/1	19.61 ± 4.51 *	13.90 ± 2.91	26.90 ± 3.93 *
*P. acidilactici* 35/1	24.82 ± 2.19 *	8.41 ± 0.80	36.25 ± 12.74 *
*P. parvulus* OK-S	26.29 ± 2.53 *	7.49 ± 0.52	17.42 ± 3.28 *
*L. brevis* KKA	27.35 ± 4.33 *	7.33 ± 2.18 *	30.37 ± 1.13 *
*L. plantarum* 8AN	26.60 ± 0.86 *	10.28 ± 0.86	18.62 ± 3.36 *
*L. salivarius* 9AN	23.82 ± 1.75 *	9.88 ± 1.23	33.18 ± 2.60 *
*L. casei* 12AN	27.27 ± 2.30 *	7.37 ± 1.45 *	20.15 ± 1.34 *
*L. plantarum* 145	24.08 ± 3.65 *	8.74 ± 1.09	21.63 ± 5.72 *
*L. acidophilus* 573	18.94 ± 3.13 *	5.49 ± 1.59 *	24.06 ± 3.64 *

## Data Availability

The data presented in this study are available in this article and are available from the corresponding authors upon reasonable request.

## Supplementary Materials

The following supporting information can be downloaded at: https://www.mdpi.com/article/10.3390/pathogens11111367/s1, Table S1: Lactic acid bacteria strains used in the conducted experiments; Table S2: Average diameters [mm] of zones of growth inhibition of microorganisms by collection lactic acid bacteria (LAB) strains (±standard deviation). Differences regarding the antimicrobial activity of the analyzed LAB were tested using the Kruskal–Wallis test (KWW test), followed by a multiple comparison test (MCT) to indicate significant differences between the groups at *p* < 0.05. The significant difference in the given strain LAB activity against pathogens is indicated with *; Table S3: Average diameters [mm] of zones of growth inhibition of microorganisms by lactic acid bacteria (LAB) strains isolated from honeybee environment of different origin (±standard deviation). Differences regarding the antimicrobial activity of the analyzed LAB were tested using the Kruskal–Wallis test (KWW test), followed by a multiple comparison test (MCT) to indicate significant differences between the groups at *p* < 0.05. The significant difference in the given strain LAB activity against pathogens is indicated with *; Table S4: Average diameters (mm) of zones of growth inhibition of microorganisms by lactic acid bacteria strains (±standard deviation), only LAB strains showing significantly increased growth inhibition zones against different pathogens. Differences regarding the antipathogenic activity of the analyzed LAB were tested using the Kruskal–Wallis test (KWW test), followed by a multiple comparison test (MCT) to indicate significant differences between the groups at *p* < 0.05. The significant difference in the given strain LAB activity against pathogens is indicated with *.
