# Supplementary material for: Antagonistic Activity of Potentially Probiotic Lactic Acid Bacteria against Honeybee (Apis mellifera L.) Pathogens
Source: Pathogens. 2022 Nov 16;11(11):1367. doi: 10.3390/pathogens11111367 (PMC9693384; doi:10.3390/pathogens11111367)
Supplement: Supplementary file 1 [file pathogens-11-01367-s001.zip › Table S1.pdf]

Table S1. Lactic acid bacteria strains used in the conducted experiments.

Isolates from honeybee environment	Source of isolation	Collection strains	Source of isolation
P. acidilactici 1/4	Large Indian cress (*Tropaeolum majus* L.)	*L. plantarum* ŁOCK 0981	Fermented cucumbers
P. acidilactici 2/1	Peony (*Peonia officinalis* L.)	*L. plantarum* ŁOCK 0982	Sourdough for sour rye soup
L. plantarum 2/2		*L. brevis* ŁOCK 0983	Fermented cucumbers
L. plantarum 3/1	European smoketree (*Cotinus coggygria* L.)	*L. brevis* ŁOCK 0984	Fermented cabbage
P. acidilactici 4/1	Black locust (*Robinia pseudoaccacia* L.)	*L. paracasei* ŁOCK 0985	Fermented cow milk
L. plantarum 5/1	Weigela (*Weigela florida* DC.)	*L. delbrueckii* ŁOCK 0987	Infant faces
P. acidilactici 5/2		*L. plantarum* ŁOCK 0989	Fermented cucumbers
P. acidilactici 6/1	Brown knapweed (*Centaurea jacea* L.)	*L. plantarum* ŁOCK 0990	Fermented cucumbers
P. pentosaceus 6/3		*L. plantarum* ŁOCK 0991	Fermented cucumbers
P. acidilactici 7/1	Flanders poppy (*Papaver rhoeas* L.)	*L. paracasei* ŁOCK 0993	Fermented cow milk
P. acidilactici 8/1	Wild mustard (*Sinapis arvensis* L.)	*L. plantarum* ŁOCK 0995	Fermented cucumbers
P. pentosaceus 8/2		*L. plantarum* ŁOCK 0996	Fermented cucumbers
L. plantarum 8/4		*L. rhamnosus* ŁOCK 0997	Infant faces
P. acidilactici 9/1	Red clover (*Trifolium pratense* L.)	*L. plantarum* 7AN	Chicken faces with dust litter
P. pentosaceus 9/3		*L. plantarum* 8AN	Chicken faces with dust litter
P. pentosaceus 10/1	Elderberry (*Sambucus nigra* L.)	*L. salivarius* 9AN	Chicken faces with dust litter
L. plantarum 10/2		*L. coryniformis* 10AN	Fermented cabbage
L. plantarum 11/1	Mock orange (*Philadelphus coronaries* L.)	*L. coryniformis* 11AN	Fermented cabbage
P. pentosaceus 11/3		*P. parvulus* OK-S	Fermented cucumbers
P. pentosaceus 12/1	Small-leaved lime 1 (*Tilia cordata* L.)	*L. plantarum* OK-B	Fermented cucumbers
P. pentosaceus 13/2	Small-leaved lime 2 (*T. cordata* L.)	*L. brevis* KKA	Fermented cabbage
P. pentosaceus 14/1	Common lavender (*Lavandula augustifolia* L.)	*L. plantarum* 145	Vegetable silage
L. plantarum 14/3		*L. brevis* W81	Sugar beet pulp
P. pentosaceus 15/1	Catalpa (*Catalpa* Scop.)	*L. rhamnosus* PL53A	nd *
L. plantarum 15/2		*L. mesenteroides* T7	Jerusalem artichoke
P. pentosaceus 16/1	Honeybee pollen	*L. plantarum* AXG KT751285	Vegetable silage
P. acidilactici 16/4		*L. mesenteroides* T5	Jerusalem artichoke
L. plantarum 17/1	Common lavender (*L. augustifolia* L.)	*L. brevis* 1	Jerusalem artichoke
P. pentosaceus 17/3		*L. brevis* P162	Sugar beet silage
L. plantarum 18/1	Butterfly bush (*Buddleja davidii* L.)	*L. farraginis* T6	Jerusalem artichoke
P. pentosaceus 19/1	Heather (*Calluna vulgaris* L.)	*L. plantarum* W12A	Sugar beet pulp
L. plantarum 20/1	Honey with the addition of other honeybee products	*L. plantarum* W	Bakery sourdough

L. plantarum 21/1	Freshly harvested *L. plantarum* 18/1 fermented honey	*L. brevis* P147	Sugar beet silage
P. acidilactici 22/2	Royal jelly	*L. plantarum* 127	Vegetable silage
P. acidilactici 23/1	Honeybee bread	*L. casei* ŁOCK 0906	Human faces
P. acidilactici 24/1	Honeydew honey	*L. plantarum* W12	Sugar beet pulp
P. acidilactici 25/1	Heather-nectar honey	*L. plantarum* 118	Vegetable silage
P. pentosaceus 26/1	Cornflower honey	*L. brevis* P1648.2	Sugar beet silage
P. acidilactici 27/1	Dandelion honey	*L. plantarum* ATCC 8014	nd
P. pentosaceus 28/1	Phacelia honey	*L. brevis* W3A	Sugar beet pulp
L. plantarum 29/1	Hawthorn honey	*L. plantarum* 150	Vegetable silage
P. pentosaceus 30/1	Forest honey	*L. plantarum* AXD KT751284	Vegetable silage
P. pentosaceus 31/1	Meadow marsh honey	*L. casei* 12AN	Human faces
P. pentosaceus 32/1	Spring honey	*L. plantarum* 120	Vegetable silage
L. plantarum 33/1	Clover honey	*L. plantarum* 113	Vegetable silage
P. pentosaceus 34/1	Lime honey	*L. acidophilus* 573	nd
P. acidilactici 35/1	Goldenrod honey	*L. plantarum* 124	Vegetable silage
P. acidilactici 36/1	Nectar honey	*L. acidophilus* LA-5	Starter culture Chr. Hansen
P. acidilactici 37/1	Coniferous honeydew honey	*L. paracasei* ŁOCK 0916	Human faces
P. pentosaceus 38/1	Melilot and meadow honey	*L. plantarum* 155	Vegetable silage
P. pentosaceus 39/1	Rape (*Brassica napus* L.)	*L. fermentum* 57A	nd
		A. kunkeei DSM 12361	Honeybee gut

* nd – no data
