# Supplementary material for: Antagonistic Activity of Potentially Probiotic Lactic Acid Bacteria against Honeybee (Apis mellifera L.) Pathogens
Source: Pathogens. 2022 Nov 16;11(11):1367. doi: 10.3390/pathogens11111367 (PMC9693384; doi:10.3390/pathogens11111367)
Supplement: Supplementary file 1 [file pathogens-11-01367-s001.zip › Table S2.pdf]

Table S2. Average diameters [mm] of zones of growth inhibition of microorganisms by collection lactic acid bacteria (LAB) strains (± standard deviation). Differences regarding the antimicrobial activity of the analyzed LAB were tested using the Kruskal–Wallis test (KWW test), followed by a multiple comparison test (MCT) to demonstrate significant differences between the groups at p < 0.05. The significant difference in the given strain LAB activity against pathogens is indicated with *.

Collection LAB strains	0981	0982	0983	0984	0985	0987	0989	0990	0991	0993	0995
P. larvae **ATCC 25367**	7.00±0.00	5.33±0.58	4.00±0.00	5.00±0.00	6.00±0.00	7.00±0.00	5.00±0.00	3.67±0.58	8.00±0.00	5.33±0.58	5.67±0.58
P. larvae **ATCC 49843**	8.00±0.00	7.00±0.00	6.33±0.58	7.33±0.58	8.00±0.00	6.00±0.00	8.00±0.00	4.00±0.00	7.00±0.00	5.00±0.00	5.00±0.00
P. apiarius **DSM 5582**	7.7±0.58	5.0±0.00	5.3±0.58	11.3±0.58	5.0±0.00	8.0±0.00	5.0±0.00	4.0±0.00	6.3±0.58	4.3±0.58	5.3±0.58
P. alvei **DSM 29**	7.00±0.00	5.00±0.00	5.00±0.00	8.67±0.58	5.67±0.58	6.67±1.15	6.33±0.58	3.00±0.00	6.00±0.00	5.00±1.00	5.00±0.00
L. sphaericus **DSM 1866**	7.00±0.00	3.00±1.00	5.67±0.58	8.00±0.00	7.67±0.58	7.00±1.00	7.00±0.00	4.00±0.00	7.00±0.00	5.00±0.00	5.00±0.00
M. plutonius **DSM 29964**	0.00	4.00±1.00	0.00	0.00	13.00±1.00	0.00 *	0.00	0.00 *	0.00	14.00±1.00	12.00±1.00
E. coli **ATCC 25922**	6.33±0.58	10.00	8.67±1.56	11.67±0.58	8.33±0.58	8.00±0.00	8.67±0.58	3.33±0.58	10.00	10.00	8.33±0.58
E. persicina **40**	7.00±1.73	21.33±1.53	20.00	19.00±0.00	20.67±1.16	20.00±3.00	16.00±1.00	17.67±2.52	14.67±0.58	15.67±0.58	20.67±0.58
P. agglomerans **43**	21.33±0.58	23.00±0.00	20.00±1.00	25.33±2.52	18.67±1.53	20.67±0.58	21.00±1.00	24.33±4.93 *	16.00±1.00	19.67±2.52	21.33±1.53
E. kobei **40**	6.00±0.00	12.33±2.08	0.00	10.67±0.58	9.00±1.00	12.33±1.16	10.00	5.67±0.58	0.00	6.67±0.58	7.00±1.00
E. cloacae **41**	22.67±0.58	23.33±2.08	0.00	7.00±0.00	21.67±0.58 *	30.00 *	23.00±0.00	17.67±2.52	18.63±0.00	23.67±1.53	26.67±0.58 *
B. faecis **DSM 24798**	10.00	0.00	0.00	0.00	10.00	7.00±1.00	0.00	10.00	0.00	10.00	7.00±1.00
B. intestinalis **DSM 17393**	0.00	0.00	7.00±1.00	0.00	0.00 *	0.00 *	0.00	0.00 *	0.00	5.00±1.00	0.00 *
**p value**					0.0310	0.0442		0.0442			0.0231

Collection LAB strain	0996	0997	7AN	8AN	9AN	10AN	11AN	OK-S	OK-B	KKA	145
***P. larvae* ATCC 25367**	9.00±0.00	8.00±1.00	5.67±0.58	7.00±0.00	7.00±1.00	6.33±0.58	6.33±0.58	8.00±1.00	7.00±0.00	5.67±0.58	8.33±0.58
***P. larvae* ATCC 49843**	6.00±0.00	7.33±0.58	7.00±0.00	6.67±0.58	8.00±0.00	8.00±0.00	5.67±0.58	6.67±0.58	7.00±0.00	8.00±0.00	8.00±0.00
***P. apiarius* DSM 5582**	10.0±0.00	8.0±0.00	6.7±0.58	5.7±0.58	9.7±0.58	6.0±0.00	5.0±0.00	13.3±2.08	7.0±0.00	6.0±0.00	10.3±0.58
***P. alvei* DSM 29**	7.00±0.00	7.00±0.00	6.00±0.00	4.67±0.58	9.00±0.00	5.67±0.58	5.00±0.00	5.67±0.58	6.00±0.00	6.00±0.00	7.67±0.58
***L. sphaericus* DSM 1866**	8.00±0.00	5.33±0.58	7.00±0.00	4.00±0.00	9.33±0.58	5.00±0.00	7.00±0.00	6.00±0.00	7.00±0.00	8.33±0.58	7.67±0.58
***M. plutonius* DSM 29964**	0.00	0.00	0.00	12.00±1.00	18.00±1.00	0.00	0.00	14.00±1.00	0.00 *	15.00±1.00	0.00±1.00 *
***E. coli* ATCC 25922**	9.00±1.00	8.33±0.58	8.00±0.00	2.00±1.00	8.00±1.00	9.00±0.00	9.00±0.00	10.00	10.00	8.00±1.00	7.00±0.00
***E. persicina* 40**	11.00±1.00	22.00±1.00	24.00±3.61	20.33±1.16	20.00	25.33±0.58	22.00±1,73	19.00±0.00	16.33±0.58	23.00±1.73	16.67±0.58
***P. agglomerans* 43**	22.00±1.00	25.67±0.58	25.00±0.00	22.33±1.54	20.67±1.54	24.67±2.52	23.67±2.08	21.67±1.53	21.33±0.58	23.67±1.53	22.33±2.52
***E. kobei* 40**	18.00±1.00	9.33±1.16	10.00	8.67±0.58	10.67±0.58	11.00±1.00	10.67±0.58	8.67±0.58	9.00±1.00	12.00±1.00	10.00
***E. cloacae* 41**	18.00±1.73	25.33±0.58	26.67±1.53	27.00±1.00 *	25.00±0.00 *	27.33±0.58	26.00±1.00	30.00±2.00 *	31.00±1.73 *	35.67±1.53 *	30.67±1.16
***B. faecis* DSM 24798**	0.00	9.00±1.00	0.00	9.00±1.00	0.00 *	10.00	0.00	6.00±0.00	2.67±0.58	0.00 *	12.00±1.00
***B. intestinalis* DSM 17393**	0.00	0.00	2.67±0.58	0.00 *	0.00 *	0.00	0.00	4.00±1.00 *	0.00 *	7.00±1.00	0.00 *
**p value**				0.0231	0.0442			0.0249	0.0442	0.0231	0.0442

Collection LAB strains	W81	573	PL53A	T7	AXG KT751285	T5	1	P162	T6	W12A	W
***P. larvae* ATCC 25367**	4.00±0.00	8.33±0.58	5.00±1.00	4.00±0.00	4.67±0.58	5.00±0.00	1.00±0.00	4.00±0.00	7.33±0.58	2.67±0.58	3.67±0.58
***P. larvae* ATCC 49843**	5.67±0.58	8.00±0.00	5.33±0.58	8.67±0.58	5.00±0.00	6.00±1.00	5.00±0.00	3.33±0.58	7.00±0.00	5.00±0.00	5.33±0.58
***P. apiarius* DSM 5582**	4.0±0.00	6.7±0.58	6.3±0.58	8.0±0.00	4.7±0.58	6.0±1.00	5.0±0.00	3.3±0.58	12.0±1.00	5.0±0.00	5.3±0.58
***P. alvei* DSM 29**	4.67±0.58	6.00±0.00	6.00±0.00	6.67±0.58	5.00±0.00	5.00±0.00	5.67±0.58	4.00±0.00	6.33±0.58	5.00±0.00	4.00±0.00
***L. sphaericus* DSM 1866**	5.67±0.58	6.00±1.00	3.00±0.00	9.67±0.58	3.00±0.00	5.00±0.00	0.00 *	2.67±0.58	7.00±0.00	4.33±0.58	4.67±0.58
***M. plutonius* DSM 29964**	12.00±1.00	10.00±1.00	12.00±1.00	0.00 *	0.00	0.00	4.00±1.00	0.00	0.00	0.00	0.00
***E. coli* ATCC 25922**	6.33±0.58	4.33±0.58	5.67±0.58	7.67±0.58	10.00	9.00±1.00	5.33±0.58	7.00±1.00	6.33±0.58	7.33±0.58	6.00±1.00
***E. persicina* 40**	4.00±0.00	21.33±0.58	22.33±2.31	10.00±0.58	20.33±0.58	4.00±0.00	20.67±1.53	20.00±1.00	6.33±0.58	7.67±0.58	19.67±0.58
***P. agglomerans* 43**	4.00±0.00	24.67±1.53	22.00±2.00	4.33±2.08	24.00±1.00	6.00±0.00	22.33±1.53	21.33±2.52	8.00±2.65	9.00±0.1.00	23.33±2.08
***E. kobei* 40**	0.00 *	9.67±1.16	10.33±0.58	0.00	11.67±0.58	0.00	6.67±0.58	11.33±1.16	3.67±0.58	7.00±0.00	7.33±0.58
***E. cloacae* 41**	4.33±2.08	31.00±2.65 *	30.67±3.79	4.00±0.00	29.67±1.16	6.67±0.58	26.67±1.16	21.67±0.58	9.33±1.16	11.33±1.16	24.33±2.31
***B. faecis* DSM 24798**	5.00±1.00	6.00±0.00	0.00 *	0.00	0.00	10.00±1.00	11.00±0.00	3.00±0.00	0.00	0.00	8.00±0.00
***B. intestinalis* DSM 17393**	0.00 *	0.00 *	0.00 *	0.00	0.00	0.00	0.00 *	0.00	0.00	0.00	0.00
**p value**	0.0442	0.0231	0.0442	0.0231			0.0442				

Collection LAB strains	P147	127	906	W12	118	P1648.2	ATCC 8014	W3A	150	AXD KT751284	12AN
P. larvae **ATCC 25367**	7.00±0.00	5.00±0.00	7.67±0.58	5.00±0.58	8.00±0.00	5.00±0.0.00	1.33±0.58	4.00±0.00	7.00±1.00	5.00±0.00	9.00±1.00
P. larvae **ATCC 49843**	5.00±0.00	8.33±0.58	8.33±0.58	5.00±0.00	7.00±0.00	4.00±0.00	6.00±1.00	5.00±1.00	6.67±0.58	4.33±0.58	7.67±0.58
P. apiarius **DSM 5582**	6.0±0.00	7.7±0.58	6.7±0.58	5.7±0.58	6.3±0.58	5.0±0.00	6.0±1.00	4.0±0.00	6.3±0.58	4.0±0.00	6.0±0.00
P. alvei **DSM 29**	5.33±0.58	7.33±1.15	8.33±0.58	5.00±0.00	6.00±0.00	4.00±0.00	5.33±0.58	4.00±0.00	6.00±0.00	4.00±0.00	6.00±0.00
L. sphaericus **DSM 1866**	5.33±0.58	6.67±0.58	6.33±0.58	5.67±0.58	5.33±0.58	4.00±1.00	5.33±0.58	3.67±0.58	5.67±0.58	2.00±1.00	5.00±1.00 *
M. plutonius **DSM 29964**	0.00	3.33±.0.58	0.00	9.00±1.00	0.00 *	0.00	10.00±1.00	0.00	15.00±1.00 *	0.00	9.00±1.00
E. coli **ATCC 25922**	7.00±0.00	7.67±0.58	7.67±1.55	8.67±0.58	10.00	6.67±0.58	7.67±0.58	8.00±0.00	8.00±0.00	8.00±1.00	7.33±0.58
E. persicina **40**	4.67±2.31	18.33±0.58	21.00±0.00	9.00±1.00	7.33±1.53	4.00±1.73	6.00±1.00	4.00±1.00	8.00±0.00	17.67±2.89	21.00±1.00
P. agglomerans **43**	3.33±0.58	21.00±2.65	21.00±0.00	9.00±1.00	10.67±0.58	6.00±0.00	6.00±0.00	6.33±2.31	10.33±0.58	22.33±2.31	21.00±1.00
E. kobei **40**	0.00	11.00±1.00	13.00±1.00	4.00±1.00	6.00±0.00	8.00±0.00	6.00±0.00	0.00	6.67±0.58	12.00±0.00	10.00±1.73
E. cloacae **41**	4.00±1.00	25.00±1.00 *	24.00±2.65	9.67±0.58	15.00±1.00	3.00±0.00	10.33±1.53	2.00±0.00	13.33±1.16	23.00±2.00	24.67±1.53 *
B. faecis **DSM 24798**	0.00	10.00	0.00	10.00	6.00±0.00	0.00	0.00	0.00	5.00±1.00	0.00	6.00±1.00
B. intestinalis **DSM 17393**	0.00	0.00 *	0.00	0.00	0.00 *	0.00	0.00	0.00	0.00 *	0.00	11.00±1.00
**p value**		0.0231			0.0442				0.0231		0.0475

Collection LAB strain	120	113	124	LA-5	916	155	57A	DSM 12361
***P. larvae* ATCC 25367**	6.00±0.00	8.00±0.00	5.00±0.00	3.67±0.58	6.00±1.00	5.00±0.00	5.00±0.00	0.00
***P. larvae* ATCC 49843**	6.33±0.58	7.00±0.00	6.00±0.00	5.00±0.00	5.00±0.00	5.00±0.00	4.67±0.58	10.00
***P. apiarius* DSM 5582**	6.0±0.00	8.0±0.00	7.7±0.58	5.3±0.58	6.0±0.00	5.0±0.00	4.3±0.58	3.00±0.00
***P. alvei* DSM 29**	6.33±0.58	7.00±0.00	6.00±0.00	5.00±0.00	5.33±0.58	6.67±0.58	3.67±0.58	6.00±0.00
***L. sphaericus* DSM 1866**	5.00±0.00	5.67±0.58	6.00±0.00	5.67±0.58	5.33±0.58	4.33±0.58	4.00±0.00	3.00±0.00
***M. plutonius* DSM 29964**	10.00±1.00	0.00	10.00±1.00	11.00±1.00 *	10.00±1.00	12.00±1.00	10.00±1.00	0.00
***E. coli* ATCC 25922**	7.00±0.00	4.00±1.00	7.67±0.58	6.00±0.00	6.33±0.58	10.00	10.00	0.00
***E. persicina* 40**	18.00±1.00	19.00±0.00	18.00±0.00	3.00±1.00	18.00±0.00	19.33±0.58	18.00±3.00	0.00
***P. agglomerans* 43**	21.33±0.58	21.67±0.58	21.33±1.53	6.00±0.00	19.33±1.16	24.33±2.89	20.00±1.00	2.00±0.00
***E. kobei* 40**	10.33±0.58	13.00±1.00	9.00±1.00	3.00±1.00	10.00	7.00±0.00	6.00±1.00	0.00
***E. cloacae* 41**	27.00±1.00	31.00±0.00	31.00±0.00 *	2.00±0.00	26.00±0.00 *	31.33±1.53 *	22.33±1.16 *	0.00
***B. faecis* DSM 24798**	11.00±1.00	0.00	10.00	10.00±1.00	10.00±1.00	9.00±1.00	5.00±1.00	0.00
***B. intestinalis* DSM 17393**	6.00±0.00	0.00	4.00±0.00 *	0.00 *	0.00 *	0.00 *	0.00 *	0.00
**p value**			0.0231	0.0310	0.0231	0.0231	0.0268	
