# Supplementary material for: Antagonistic Activity of Potentially Probiotic Lactic Acid Bacteria against Honeybee (Apis mellifera L.) Pathogens
Source: Pathogens. 2022 Nov 16;11(11):1367. doi: 10.3390/pathogens11111367 (PMC9693384; doi:10.3390/pathogens11111367)
Supplement: Supplementary file 1 [file pathogens-11-01367-s001.zip › Table S3.pdf]

Table S3. Average diameters [mm] of zones of growth inhibition of microorganisms by lactic acid bacteria (LAB) strains isolated from honeybee environment of different origin (± standard deviation). Differences regarding the antimicrobial activity of the analyzed LAB were tested using the Kruskal–Wallis test (KWW test), followed by a multiple comparison test (MCT) to demonstrate significant differences between the groups at $p < 0.05$. The significant difference in the given strain LAB activity against pathogens is indicated with *.

LAB strains isolated from honeybee environment	1/4	2/1	2/2	3/1	4/1	5/1	5/2	6/1	6/3	7/1	8/1
P. larvae **ATCC 25367**	16.44±0.58	16.78±0.58	12.22±0.58	14.67±1.00	15.56±0.58	13.56±0.58	15.00±0.58	12.67±0.00	14.22±0.58	16.00±0.58	9.67±1.00
P. larvae **ATCC 49843**	8.00±0.00	7.67±0.58	7.00±1.00	8.00±0.00	12.00±0.00	8.00±0.00	10.00	9.67±0.58	8.00±0.00	8.33±1.53	9.33±1.55
P. apiarius **DSM 5582**	31.67±2.52	39.67±0.58	33.33±0.58	35.67±2.08	30.67±0.58	37.33±2.31	32.00±2.00	33.33±0.58 *	28.67±1.15	31.33±0.58 *	32.00±1.00
P. alvei **DSM 29**	30.67±4.16	29.67±0.58	30.67±0.58	31.67±2.89	36.67±5.03 *	32.00±0.00	32.00±1.00	29.67±1.16	32.00±1.00 *	27.00±2.65	28.33±3.51
L. sphaericus **DSM 1866**	3.67±1.53	4.67±0.58	6.67±1.53	4.33±1.53	8.67±2.08	4.33±2.08	6.33±2.31	4.33±1.53	2.00±1.00	6.67±1.16	8.00±1.00
M. plutonius **DSM 29964**	12.67±0.58	0.00	0.00	0.00	11.00±1.00	0.00	0.00	11.00±0.00	11.00±0.00	10.33±0.58	10.67±0.58
E. coli **ATCC 25922**	6.33±0.58	4.67±0.58	6.00±1.00	5.00±0.00	5.00±0.00	5.33±0.58	5.33±0.58	8.67±0.58	6.00±1.00	9.00±0.00	8.00±0.00
E. persicina **40**	12.33±0.58	9.67±0.58	10.33±0.58	10.00±1.00	11.67±1.53	8.67±1.53	11.33±3.22	6.33±0.1.16	9.33±1.16	9.00±1.00	10.00
P. agglomerans **43**	12.00±0.00	14.67±0.58	14.33±0.58	11.00±1.00	10.00	10.00	10.67±1.16	9.33±0.58	13.67±0.58	14.00±0.00	9.00±0.00
E. kobei **40**	25.00±0.00	26.00±0.00	12.00±0.00	23.00±0.00	25.00±0.00	22.00±0.00	23.00±1.73	22.33±0.58	19.67±0.58	25.00±0.00	10.00
E. cloacae **41**	8.67±1.16	10.00	10.00	8.67±1.16	9.67±0.58	10.00	9.00±2.00	9.33±1.16	7.33±0.58	8.00±1.00	11.00±0.00
B. faecis **DSM 24798**	0.00	0.00	0.00	0.00	0.00 *	0.00	0.00	0.00 *	0.00 *	0.00 *	0.00
B. intestinalis **DSM 17393**	0.00	0.00	0.00	0.00	0.00 *	0.00	0.00	0.00 *	0.00 *	0.00 *	0.00
**p value (KWW test)**					0.0442			0.0442	0.0442	0.0442	

LAB strains isolated from honeybee environment	8/2	8/4	9/1	9/3	10/1	10/2	11/1	11/3	12/1	13/2	14/1
***P. larvae* ATCC 25367**	11.00±1.00	16.11±0.58	16.89±0.58	16.44±0.58	12.89±0.58	12.67±0.00	14.22±1.00	13.89±1.00	9.33±0.58	8.89±0.00	11.33±0.58
***P. larvae* ATCC 49843**	10.00	5.33±1.55	7.33±0.58	7.67±0.58	8.67±0.58	9.67±0.58	11.00±0.00	10.67±0.58	7.00±1.00	10.67±0.58	9.67±0.58
***P. apiarius* DSM 5582**	30.67±1.15	38.33±1.15 *	32.33±2.31	30.67±0.58	24.00±1.00	32.33±0.58	30.00±1.00	32.67±2.52	30.67±1.15	34.33±2.08 *	35.67±0.58 *
***P. alvei* DSM 29**	30.67±2.09	28.00±2.65	32.33±1.16	33.67±10.97	27.33±2.08 *	31.33±3.06	26.33±2.08	30.33±0.58	27.00±1.00	26.33±1.53	28.33±0.58
***L. sphaericus* DSM 1866**	4.33±1.53	3.33±0.58	6.67±0.58	4.67±1.16	4.33±0.58	3.33±0.58	4.67±0.58	4.33±0.58	2.00±1.00	2.33±0.58	3.33±0.58
***M. plutonius* DSM 29964**	10.67±0.58	10.00±1.00	10.00±1.00	10.67±0.58	8.00±1.00	10.00	0.00	0.00	0.00	10.00	10.00
***E. coli* ATCC 25922**	9.00±0.00	3.33±0.58	8.67±0.58	11.67±0.58	7.67±0.58	8.00±0.00	5.67±0.58	9.33±0.58	5.67±0.58	6.00±0.00	5.00±1.00
***E. persicina* 40**	11.00±0.00	13.00±1.00	12.00±0.00	10.33±0.58	6.67±0.58	9.33±0.58	9.33±0.58	10.00±1.00	6.33±0.58	7.33±0.58	10.33±1.53
***P. agglomerans* 43**	12.00±1.00	13.67±1.16	13.67±1.16	9.00±1.00	9.33±1.16	9.00±0.00	11.00±1.00	6.33±1.16	10.67±0.58	9.00±0.00	9.67±0.58
***E. kobei* 40**	10.00	21.67±2.08	25.00±1.00	30.00	22.67±1.16	19.67±0.58	22.33±0.58	25.33±0.58	11.00±1.00	10.33±0.58	14.00±0.00
***E. cloacae* 41**	11.00±1.00	7.33±0.58	10.33±0.58	10.00	10.00	9.33±0.58	10.67±0.58	8.33±0.58	9.00±0.00	8.33±0.58	9.67±1.53
***B. faecis* DSM 24798**	0.00	0.00 *	0.00	0.00	0.00 *	0.00	0.00	0.00	0.00	0.00 *	0.00 *
***B. intestinalis* DSM 17393**	0.00	0.00 *	0.00	0.00	0.00 *	0.00	0.00	0.00	0.00	0.00 *	0.00 *
**p value**		0.0442			0.0442					0.0442	0.0442

LAB strains isolated from honeybee environment	14/3	15/1	15/2	16/1	16/4	17/1	17/3	18/1	19/1	20/1	21/1
P. larvae **ATCC 25367**	12.11±0.58	12.67±0.58	15.22±0.00	12.67±0.58	11.11±0.00	13.89±0.58	14.89±1.53	14.22±0.58	14.78±0.58	14.33±0.58	14.44±0.58
P. larvae **ATCC 49843**	8.33±0.58	7.33±0.58	8.00±0.00	5.00±1.00	7.00±0.00	8.67±0.58	8.67±1.53	8.00±1.00	6.67±0.58	7.00±0.00	9.33±0.58
P. apiarius **DSM 5582**	30.67±1.15	32.33±1.15	35.00±3.61	33.00±1.00	43.33±1.53 *	29.00±1.00	32.67±0.58	30.00	33.00±1.00	34.33±0.58 *	35.33±4.16
P. alvei **DSM 29**	29.67±0.58	29.00±1.00	32.33±0.58	27.00±2.65	31.33±1.53	29.33±3.06	31.00±1.00	32.67±0.58 *	31.00±1.73	28.67±1.16	32.00±2.00
L. sphaericus **DSM 1866**	5.00±2.00	5.33±0.58	3.33±0.58	6.00±0.00	1.33±0.58	4.33±0.58	4.33±2.31	6.67±1.16	8.00±2.65	6.33±1.53	6.00±1.00
M. plutonius **DSM 29964**	6.67±0.58	0.00	0.00	0.00	9.67±0.58	9.33±0.58	0.00	10.00±1.00	13.00±1.00	12.67±0.58	14.33±0.58
E. coli **ATCC 25922**	4.67±0.58	6.00±0.00	5.33±0.58	4.00±1.00	7.00±0.00	5.00±0.00	5.67±0.58	5.00±0.00	5.67±0.58	4.33±0.58	5.00±0.00
E. persicina **40**	10.33±0.58	9.67±0.58	11.33±1.53	11.00±0.00	5.00±2.65	8.00±0.00	10.33±0.58	11.33±0.58	8.67±0.58	10.33±0.58	10.00
P. agglomerans **43**	11.00±1.00	9.33±0.58	11.33±0.58	7.00±0.00	7.67±0.58	12.00±0.00	11.33±0.58	8.00±0.00	12.00±1.00	11.00±0.00	8.00±0.00
E. kobei **40**	15.00±0.00	19.00±1.73	23.00±1.73	20.00±1.00	20.67±0.58	21.67±0.58	23.00±1.00	23.33±1.53	23.67±1.53	21.67±2.89	25.33±4.51
E. cloacae **41**	7.00±0.00	7.00±1.00	9.33±0.58	6.33±0.58	8.00±0.00	9.67±0.58	10.67±0.58	9.33±0.58	9.33±0.58	6.00±0.00	11.33±0.58
B. faecis **DSM 24798**	0.00	0.00	0.00	0.00	0.00 *	0.00	0.00	0.00 *	0.00	0.00 *	0.00
B. intestinalis **DSM 17393**	0.00	0.00	0.00	0.00	0.00 *	0.00	0.00	0.00 *	0.00	0.00 *	0.00
**p value**					0.0442			0.0442		0.0442	

LAB strains isolated from honeybee environment	22/1	23/1	24/1	25/1	26/1	27/1	28/1	29/1	30/1	31/1	32/1
P. larvae **ATCC 25367**	13.00±0.58	14.44±0.00	11.22±0.58	14.56±0.58	15.78±1.15	15.22±0.00	11.89±0.58	13.44±0.58	11.78±0.58	11.67±0.58	10.56±0.58
P. larvae **ATCC 49843**	7.33±0.58	6.00±1.00	7.00±1.00	7.67±0.58	5.00±0.00	6.67±0.58	6.00±2.00	7.00±1.00	5.67±0.58	6.00±0.00	8.33±0.58
P. apiarius **DSM 5582**	27.33±2.08	30.67±1.15	29.33±1.15	27.33±2.52	32.33±2.08	29.33±1.15	27.33±2.52	36.00±3.61 *	31.33±1.53	26.67±1.55	32.33±1.55
P. alvei **DSM 29**	26.67±2.08	29.67±0.58	29.00±0.00	28.67±1.16	31.00±1.73	29.00±1.00	26.33±2.31	31.00±1.73	30.00	28.00±1.73	31.33±2.31
L. sphaericus **DSM 1866**	4.00±1.00	5.67±0.58	5.33±0.58	8.00±1.00	3.33±0.58	7.00±1.00	6.00±0.00	7.00±1.00	6.33±0.58	1.67±0.58	3.00±0.00
M. plutonius **DSM 29964**	11.67±0.58	7.00±1.00	12.00±0.00	10.00	0.00	0.00	9.00±1.00	12.00±0.00	10.00	0.00	12.00±0.00
E. coli **ATCC 25922**	6.33±0.58	6.00±0.00	5.00±0.00	5.00±1.00	9.67±0.58	8.67±0.58	5.00±0.00	6.00±0.00	6.00±1.00	6.33±0.58	8.00±0.00
E. persicina **40**	8.00±1.00	7.67±0.58	10.00	11.00±1.00	10.33±1.53	12.00±0.00	11.00±0.00	9.67±0.58	10.33±0.58	10.00	9.00±1.00
P. agglomerans **43**	7.67±1.16	10.00±1.00	13.67±0.58	12.00±1.00	11.33±0.58	13.33±0.58	8.67±0.58	10.67±1.16	6.00±1.00	7.67±0.58	5.33±0.58
E. kobei **40**	23.33±2.08	25.67±4.04	10.00±1.00	20.67±2.31	25.67±0.58	20.33±1.16	16.00±0.00	20.00±1.00	19.00±1.73	17.33±1.16	17.33±1.16
E. cloacae **41**	8.67±1.53	9.67±0.58	10.67±1.16	9.33±0.58	7.33±0.58	7.67±0.58	8.67±0.58	10.00	9.67±1.16	6.33±0.58	7.00±0.00
B. faecis **DSM 24798**	0.00	0.00	0.00	0.00	0.00	0.00	0.00	0.00 *	0.00	0.00	0.00
B. intestinalis **DSM 17393**	0.00	0.00	0.00	0.00	0.00	0.00	0.00	0.00 *	0.00	0.00	0.00
**p value**								0.0442			

LAB strains isolated from honeybee environment	33/1	34/1	35/1	36/1	37/1	38/1	39/1	57A
P. larvae **ATCC 25367**	5.44±1.00	13.22±0.58	14.56±0.58	16.44±1.15	10.67±0.58	9.78±0.58	10.00±1.53	5.00±0.00
P. larvae **ATCC 49843**	5.67±0.58	5.00±0.00	8.00±1.00	6.33±0.58	7.67±0.58	6.33±0.58	7.33±0.58	4.67±0.58
P. apiarius **DSM 5582**	35.33±0.58	32.33±0.58	27.67±0.58 *	30.00±2.00	30.67±0.58	37.67±2.52	35.67±3.06	4.3±0.58
P. alvei **DSM 29**	31.67±1.53	33.00±0.00	25.33±0.58	26.33±1.53	31.67±1.53	28.00±2.00	32.67±2.08	3.67±0.58
L. sphaericus **DSM 1866**	4.67±0.58	3.33±1.16	2.67±1.16	1.00±0.00	3.33±1.16	4.33±1.16	4.00±±0.00	4.00±0.00
M. plutonius **DSM 29964**	10.00	7.33±0.58	4.33±0.58	6.67±0.58	7.67±0.58	0.00	0.00	10.00±1.00
E. coli **ATCC 25922**	6.00±0.00	8.33±1.53	10.00	10.00	4.67±0.58	10.00	8.00±0.00	10.00
E. persicina **40**	9.00±0.00	8.67±1.53	9.33±0.58	12.67±2.52	8.00±0.00	8.00±0.00	10.33±0.58	18.00±3.00
P. agglomerans **43**	11.33±0.58	10.33±0.58	13.00±0.00	13.00±0.00	13.00±0.00	13.00±0.00	10.00±1.00	20.00±1.00
E. kobei **40**	5.00±13.23	20.67±1.16	21.33±1.16	23.67±2.52	11.00±`.73	8.33±1.53	9.67±1.53	6.00±1.00
E. cloacae **41**	2.00±0.00	7.00±0.00	7.67±1.16	7.33±0.58	9.00±1.00	1.00±0.00	1.67±0.58	22.33±1.16 *
B. faecis **DSM 24798**	0.00	0.00	0.00 *	0.00	0.00	0.00	0.00	5.00±1.00
B. intestinalis **DSM 17393**	0.00	0.00	0.00 *	0.00	0.00	0.00	0.00	0.00 *
**p value**			0.0442					0.0268
