# Supplementary material for: Antagonistic Activity of Potentially Probiotic Lactic Acid Bacteria against Honeybee (Apis mellifera L.) Pathogens
Source: Pathogens. 2022 Nov 16;11(11):1367. doi: 10.3390/pathogens11111367 (PMC9693384; doi:10.3390/pathogens11111367)
Supplement: Supplementary file 1 [file pathogens-11-01367-s001.zip › Table S4.pdf]

Table S4. Average diameters (mm) of zones of growth inhibition of microorganisms by lactic acid bacteria strains (± standard deviation), only LAB strains showing significantly increased growth inhibition zones against different pathogens. Differences regarding the antipathogenic activity of the analyzed LAB were tested using the Kruskal–Wallis test (KWW test), followed by a multiple comparison test (MCT) to indicate significant differences between the groups at $p < 0.05$. The significant difference on the given strain LAB activity against pathogens is indicated with *.

LAB strains isolated from honeybee environment	4/1	6/1	6/3	7/1	8/4	10/1	13/2	14/1	16/4	18/1	20/1	29/1	35/1
P. larvae **ATCC 25367**	15.56±0.58	12.67±0.00	14.22±0.58	16.00±0.58	16.11±0.58	12.89±0.58	8.89±0.00	11.33±0.58	11.11±0.00	14.22±0.58	14.33±0.58	13.44±0.58	14.56±0.58
P. larvae **ATCC 49843**	12.00±0.00	9.67±0.58	8.00±0.00	8.33±1.53	5.33±1.55	8.67±0.58	10.67±0.58	9.67±0.58	7.00±0.00	8.00±1.00	7.00±0.00	7.00±1.00	8.00±1.00
P. apiarius **DSM 5582**	30.67±0.58	33.33±0.58 *	28.67±1.15	31.33±0.58 *	38.33±1.15 *	24.00±1.00	34.33±2.08 *	35.67±0.58 *	43.33±1.53 *	30.00	34.33±0.58 *	36.00±3.61 *	27.67±0.58 *
P. alvei **DSM 29**	36.67±5.03 *	29.67±1.16	32.00±1.00 *	27.00±2.65	28.00±2.65	27.33±2.08 *	26.33±1.53	28.33±±0.58	31.33±1.53	32.67±0.58 *	28.67±1.16	31.00±1.73	25.33±0.58
L. sphaericus **DSM 1866**	8.67±2.08	4.33±1.53	2.00±1.00	6.67±1.16	3.33±0.58	4.33±0.58	2.33±0.58	3.33±0.58	1.33±0.58	6.67±1.16	6.33±1.53	7.00±1.00	2.67±1.16
M. plutonius **DSM 29964**	11.00±1.00	11.00±0.00	11.00±0.00	10.33±0.58	10.00±1.00	8.00±1.00	10.00	10.00	9.67±0.58	10.00±1.00	12.67±0.58	12.00±0.00	4.33±0.58
E. coli **ATCC 25922**	5.00±0.00	8.67±0.58	6.00±1.00	9.00±0.00	3.33±0.58	7.67±0.58	6.00±0.00	5.00±1.00	7.00±0.00	5.00±0.00	4.33±0.58	6.00±0.00	10.00
E. persicina **40**	11.67±1.53	6.33±0.1.16	9.33±1.16	9.00±1.00	13.00±1.00	6.67±0.58	7.33±0.58	10.33±1.53	5.00±2.65	11.33±0.58	10.33±0.58	9.67±0.58	9.33±0.58
P. agglomerans **43**	10.00	9.33±0.58	13.67±0.58	14.00±0.00	13.67±1.16	9.33±1.16	9.00±0.00	9.67±0.58	7.67±0.58	8.00±0.00	11.00±0.00	10.67±1.16	13.00±0.00
E. kobei **40**	25.00±0.00	22.33±0.58	19.67±0.58	25.00±0.00	21.67±2.08	22.67±1.16	10.33±0.58	14.00±0.00	20.67±0.58	23.33±1.53	21.67±2.89	20.00±1.00	21.33±1.16
E. cloacae **41**	9.67±0.58	9.33±1.16	7.33±0.58	8.00±1.00	7.33±0.58	10.00	8.33±0.58	9.67±1.53	8.00±0.00	9.33±0.58	6.00±0.00	10.00	7.67±1.16
B. faecis **DSM 24798**	0.00 *	0.00 *	0.00 *	0.00 *	0.00 *	0.00 *	0.00 *	0.00 *	0.00 *	0.00 *	0.00 *	0.00 *	0.00 *
B. intestinalis **DSM 17393**	0.00 *	0.00 *	0.00 *	0.00 *	0.00 *	0.00 *	0.00 *	0.00 *	0.00 *	0.00 *	0.00 *	0.00 *	0.00 *
p value (KWW test)	0.0442	0.0442	0.0442	0.0442	0.0442	0.0442	0.0442	0.0442	0.0442	0.0442	0.0442	0.0442	0.0442

Collection LAB strains	0985	0987	0990	0995	8AN	9AN	OK-S	OK-B	KKA	145	W81	573
P. larvae **ATCC 25367**	6.00±0.00	7.00±0.00	3.67±0.58	5.67±0.58	7.00±0.00	7.00±1.00	8.00±1.00	7.00±0.00	5.67±0.58	8.33±0.58	4.00±0.00	8.33±0.58
P. larvae **ATCC 49843**	8.00±0.00	6.00±0.00	4.00±0.00	5.00±0.00	6.67±0.58	8.00±0.00	6.67±0.58	7.00±0.00	8.00±0.00	8.00±0.00	5.67±0.58	8.00±0.00
P. apiarius **DSM 5582**	5.0±0.00	8.0±0.00	4.0±0.00	5.3±0.58	5.7±0.58	9.7±0.58	13.3±2.08	7.0±0.00	6.0±0.00	10.3±0.58	4.0±0.00	6.7±0.58
P. alvei **DSM 29**	5.67±0.58	6.67±1.15	3.00±0.00	5.00±0.00	4.67±0.58	9.00±0.00	5.67±0.58	6.00±0.00	6.00±0.00	7.67±0.58	4.67±0.58	6.00±0.00
L. sphaericus **DSM 1866**	7.67±0.58	7.00±1.00	4.00±0.00	5.00±0.00	4.00±0.00	9.33±0.58	6.00±0.00	7.00±0.00	8.33±0.58	7.67±0.58	5.67±0.58	6.00±1.00
M. plutonius **DSM 29964**	13.00±1.00	0.00 *	0.00 *	12.00±1.00	12.00±1.00	18.00±1.00	14.00±1.00	0.00 *	15.00±1.00	0.00±1.00 *	12.00±1.00	10.00±1.00
E. coli **ATCC 25922**	8.33±0.58	8.00±0.00	3.33±0.58	8.33±0.58	2.00±1.00	8.00±1.00	10.00	10.00	8.00±1.00	7.00±0.00	6.33±0.58	4.33±0.58
E. persicina **40**	20.67±1.16	20.00±3.00	17.67±2.52	20.67±0.58	20.33±1.16	20.00	19.00±0.00	16.33±0.58	23.00±1.73	16.67±0.58	4.00±0.00	21.33±0.58
P. agglomerans **43**	18.67±1.53	20.67±0.58	24.33±4.93 *	21.33±1.53	22.33±1.54	20.67±1.54	21.67±1.53	21.33±0.58	23.67±1.53	22.33±2.52	4.00±0.00	24.67±1.53
E. kobei **40**	9.00±1.00	12.33±1.16	5.67±0.58	7.00±1.00	8.67±0.58	10.67±0.58	8.67±0.58	9.00±1.00	12.00±1.00	10.00	0.00 *	9.67±1.16
E. cloacae **41**	21.67±0.58 *	30.00 *	17.67±2.52	26.67±0.58 *	27.00±1.00 *	25.00±0.00 *	30.00±2.00 *	31.00±1.73 *	35.67±1.53 *	30.67±1.16 *	4.33±2.08	31.00±2.65 *
B. faecis **DSM 24798**	10.00	7.00±1.00	10.00	7.00±1.00	9.00±1.00	0.00 *	6.00±0.00	2.67±0.58	0.00 *	12.00±1.00	5.00±1.00	6.00±0.00
B. intestinalis **DSM 17393**	0.00 *	0.00 *	0.00 *	0.00 *	0.00 *	0.00 *	4.00±1.00 *	0.00 *	7.00±1.00	0.00 *	0.00 *	0.00 *
**p value (KWW test)**	0.0310	0.0442	0.0442	0.0231	0.0231	0.0442	0.0249	0.0442	0.0231	0.0442	0.0442	0.0231

Collection LAB strains	PL53A	T7	1	127	118	150	12AN	124	LA-5	916	155	57A
***P. larvae* ATCC 25367**	5.00±1.00	4.00±0.00	1.00±0.00	5.00±0.00	8.00±0.00	7.00±1.00	9.00±1.00	5.00±0.00	3.67±0.58	6.00±1.00	5.00±0.00	5.00±0.00
***P. larvae* ATCC 49843**	5.33±0.58	8.67±0.58	5.00±0.00	8.33±0.58	7.00±0.00	6.67±0.58	7.67±0.58	6.00±0.00	5.00±0.00	5.00±0.00	5.00±0.00	4.67±0.58
***P. apiarius* DSM 5582**	6.3±0.58	8.0±0.00	5.0±0.00	7.7±0.58	6.3±0.58	6.3±0.58	6.0±0.00	7.7±0.58	5.3±0.58	6.0±0.00	5.0±0.00	4.3±0.58
***P. alvei* DSM 29**	6.00±0.00	6.67±0.58	5.67±0.58	7.33±1.15	6.00±0.00	6.00±0.00	6.00±0.00	6.00±0.00	5.00±0.00	5.33±0.58	6.67±0.58	3.67±0.58
***L. sphaericus* DSM 1866**	3.00±0.00	9.67±0.58	0.00 *	6.67±0.58	5.33±0.58	5.67±0.58	5.00±1.00 *	6.00±0.00	5.67±0.58	5.33±0.58	4.33±0.58	4.00±0.00
***M. plutonius* DSM 29964**	12.00±1.00	0.00 *	4.00±1.00	3.33±.0.58	0.00 *	15.00±1.00 *	9.00±1.00	10.00±1.00	11.00±1.00 *	10.00±1.00	12.00±1.00	10.00±1.00
***E. coli* ATCC 25922**	5.67±0.58	7.67±0.58	5.33±0.58	7.67±0.58	10.00	8.00±0.00	7.33±0.58	7.67±0.58	6.00±0.00	6.33±0.58	10.00	10.00
***E. persicina* 40**	22.33±2.31	10.00±0.58 *	20.67±1.53	18.33±0.58	7.33±1.53	8.00±0.00	21.00±1.00	18.00±0.00	3.00±1.00	18.00±0.00	19.33±0.58	18.00±3.00
***P. agglomerans* 43**	22.00±2.00	4.33±2.08	22.33±1.53	21.00±2.65	10.67±0.58	10.33±0.58	21.00±1.00	21.33±1.53	6.00±0.00	19.33±1.16	24.33±2.89	20.00±1.00
***E. kobei* 40**	10.33±0.58	0.00	6.67±0.58	11.00±1.00	6.00±0.00	6.67±0.58	10.00±1.73	9.00±1.00	3.00±1.00	10.00	7.00±0.00	6.00±1.00
***E. cloacae* 41**	30.67±3.79	4.00±0.00	26.67±1.16 *	25.00±1.00 *	15.00±1.00 *	13.33±1.16	24.67±1.53 *	31.00±0.00 *	2.00±0.00	26.00±0.00 *	31.33±1.53 *	22.33±1.16 *
***B. faecis* DSM 24798**	0.00 *	0.00	11.00±0.00	10.00	6.00±0.00	5.00±1.00	6.00±1.00	10.00	10.00±1.00	10.00±1.00	9.00±1.00	5.00±1.00
***B. intestinalis* DSM 17393**	0.00 *	0.00	0.00 *	0.00 *	0.00 *	0.00 *	11.00±1.00	4.00±0.00 *	0.00 *	0.00 *	0.00 *	0.00 *
**p value (KWW test)**	0.0442	0.0231	0.0442	0.0231	0.0442	0.0231	0.0475	0.0231	0.0310	0.0231	0.0231	0.0268
